# Zein Nanoparticles Containing Arginine-Based Surfactants: Physicochemical Characterization and Effect on the Biological Properties

**DOI:** 10.3390/ijms24032568

**Published:** 2023-01-29

**Authors:** Lourdes Pérez, Adrià Sentís, Zakaria Hafidi, Aurora Pinazo, Maria Teresa García, Manuel Martín-Pastor, Francisco Fábio Oliveira de Sousa

**Affiliations:** 1 Department of Surfactants and Nanobiotechnology, Institute for Advanced Chemistry of Catalonia (IQAC-CSIC), 08034 Barcelona, Spain; 2 While was at Biocompatible Surfactant and Liquid Ionic Group, Institut de Química Avançada de Catalunya-CSIC, 08034 Barcelona, Spain; 3 Unidad de Resonancia Magnética, Área de Infraestructuras de Investigación, Universidad de Santiago de Compostela, 15782 Santiago de Compostela, Spain

**Keywords:** amino acid surfactants, zein, nanoparticles, antimicrobial, hemolytic, interaction, monolayer, nuclear magnetic ressonance, docking

## Abstract

Cationic surfactants carry antimicrobial activity, based on their interaction and disruption of cell membranes. Nonetheless, their intrinsic toxicity limits their applicability. To overcome this issue, a feasible strategy consists of using solid nanoparticles to improve their delivery. The zein nanoparticles were loaded with four cationic arginine-based surfactants: one single chain Nα-lauroyl-arginine (LAM) and three Gemini surfactants Nα Nω-Bis (Nα-lauroyl-arginine) α, ω—diamide) (C_3_(LA)_2_, C_6_(LA)_2_ and C_9_(LA)_2_). Blank and loaded zein nanoparticles were characterized in terms of size, polydispersity and zeta potential. Furthermore, the antimicrobial activity against bacteria and yeasts and the hemolytic activity were investigated and compared to the surfactants in a solution. Nanoparticles were found to be monodisperse, presenting a size of between 180–341 nm, a pdI of <0.2 and a positive zeta potential of between +13 and +53 mV, remaining stable over 365 days. The nanoencapsulation maintained the antimicrobial activity as unaltered, while the extensive hemolytic activity found for the surfactants in a solution was reduced drastically. Nuclear Magnetic Ressonance (NMR), molecular docking and monolayer findings indicated that zein entraps the surfactants, interfering in the surfactant–membrane interactions. Accordingly, the nanoepcasulation of arginine surfactants improved their selectivity, while the cationic charges were free to attack and destroy bacteria and fungi; the aliphatic chains were not available to disrupt the cellular membranes.

## 1. Introduction

Amino acid based cationic surfactants are a good alternative to conventional cationic surfactants, as they are biodegradable, prepared from renewable raw materials and also present low-toxic effects [1].

Cationic gemini surfactants offer a different possibility for interacting with the cellular membranes of microorganisms [2]. These surfactants carry two polar heads and two fatty chains resulting from connecting with a spacer chain two single chain cationic surfactants. Their physicochemical properties make these molecules easily absorbed within solid–liquid interphases, while the flexible structure can modify their properties [3].

One of the major sanitary concerns is the rise of resistant pathogens. Uncommon species of yeasts such as *Candida tropicalis*, *Candida glabrata* and more recently the worrisome *Candida auris* have been spreading over the last years. The number of antibiotic resistant bacteria has also been responsible for a rapid increment in infectious caused mortality. All these factors reinforce the urgent search for new potential treatments.

An effective strategy to meet today’s demand to heal a number of infections like pulmonary, skin or wounds, is to use cationic surfactants with antimicrobial properties. Those surfactants are molecules with an amphiphilic structure, containing hydrophilic and hydrophobic domains. Their structure determines the physicochemical and biological properties, being determinant on their biomedical applicability. Cationic surfactants are extensively used in a number of fields, although they present an intrinsic toxicity which limits their applicability in medical treatments. Arginine-based cationic surfactants have shown great potential as antimicrobials because of their ability to interact with the negatively charged cell membranes [4], provoking their permeabilization and the extravasation of the intracellular content. This process occurs in two phases: First, the hydrophilic positively-charged region interacts with the negatively-charged molecules of the membrane. Then, the hydrophobic region interacts with the lipid bilayer. This mechanism of action limits the microorganisms’ resistance to surfactants.

Moreover, their biocide effect was found to be more pronounced against bacteria than yeasts [5,6]. This difference can be attributed to the differences found in their cell wall composition. While the cell wall of yeasts is composed mainly of molecules with positive electrostatic balance, bacteria show negatively charged molecules in their surface: lipoteichoic acid for Gram positive and lipopolysaccharides for Gram negative strains. As a result, cationic molecules, such as surfactants, can interact easier with bacteria than yeasts. Despite these favorable properties, their activity, in some cases, is also limited by their limited solubility in water over the critical micellar concentration (CMC) [7].

Polymeric nanoparticles (PNPs) have shown some unique properties when designed for drug delivery, such as drug protection, controlled release, selective/enhanced activity and targeting. The PNPs´ structure is often designed to enable delivery to target cells or to reach metabolic pathways.

Biopolymers are among the most advantageous materials to formulate nanocarriers, due to their superior biocompatibility and biodegradability. Zein, a prolamine found in the endosperm of maize (*Zea mays*), presents the ability to entrap and carry therapeutic molecules. This protein can encapsulate both hydrophilic and hydrophobic molecules and is listed in the Generally Recognized as Safe (GRAS) FDA list. Recently, it has been used in the composition of different delivery systems, demonstrating its versatility as a nanocarrier [8,9,10,11,12,13]. These studies have shown that zein can modulate the release of their nanoparticles´ content in a non-aqueous environment such as simulated gastric and intestinal fluid, which allows the nanoparticles’ content to resist adverse conditions such as acid and basic pH [12].

The use of nanotechnology is currently studied for several purposes in the medical field, including both the enhanced antimicrobial activity associated with a reduction in adverse effects [14,15]. A large range of materials have been used in the composition of drug delivery systems, while biopolymers present some advantages, such as: biocompatibility, biodegradability, low cost, easy handling and tolerable regulatory aspects.

Zein was used in this study to prepare arginine-based nanoparticles. Zein presents key functional properties such as hydrophobicity, which facilitates the spontaneous formation of nanoparticles and the remarkable capability of carrying different molecules, such as drugs, genetic material and antigens [12] and in our case amino acid-based surfactants, allowing us obtaining nanoparticles with favorable properties.

Despite the great effectiveness against microorganisms, amino-acid derived surfactants can also interact indiscriminately with the lipid bilayer of cell membranes, making them susceptible to toxicity. This is especially important with Gemini surfactants, as the gain in the antimicrobial activity is unbalanced with a higher hemolytic activity. This is still a major limitation for new biodegradable and biocompatible surfactants [15,16,17].

**Figure 1:**
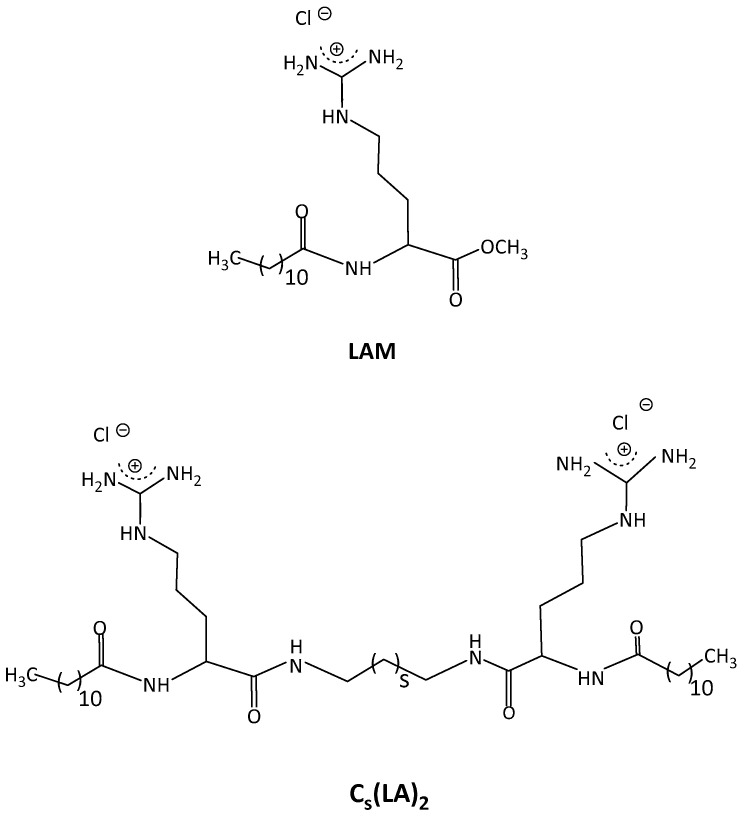
Chemical structure of the arginine-based surfactants. Single chain surfactant, LAM, Gemini surfactants s = 1 C_3_(LA)_2_, s = 4 C_6_(LA)_2_ and s = 7 C_9_(LA)_2_.

Therefore, in this paper, arginine-based surfactants (Figure 1) were loaded in the zein nanoparticles and evaluated in terms of antimicrobial and hemolytic activity in order to determine their selectivity. In addition, the interactions between zein and arginine-based surfactants have been investigated. Accordingly, the encapsulation of these surfactants in nanoparticles could reduce their hemolytic properties, thanks to the entrapment and controlled release, while their antimicrobial properties could be maintained [18].

## 2. Results and Discussion

### 2.1. Nanoparticles Formation and Characterization

The arginine-based surfactants together with the zein promote a favorable balance between effectiveness, cost, simple manufacturing and preservation. These criteria are of high importance in view of two important factors: the increasing nosocomial infections and the raising of microbial resistance to conventional treatments.

**Figure 2:**
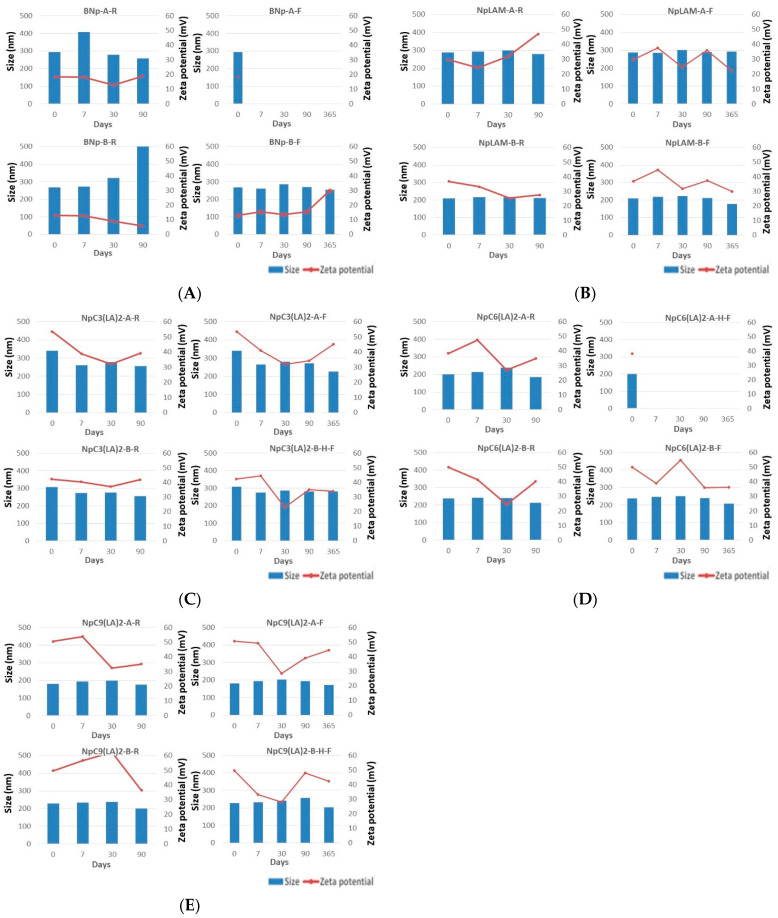
Size (nm) and zeta potential (mV) of: (**A**) blank, (**B**) LAM, (**C**) C_3_(LA)_2_, (**D**) C_6_(LA)_2_ and (**E**) C_9_(LA)_2_ loaded zein nanoparticles prepared by methods (**A**,**B**) and stored at room temperature (R) and freezer (F) over 365 days.

Satisfactory results were obtained from the proposed preparation methods: method A and method B. The nanoparticles were characterized in terms of size, pdI, zeta potential and stability immediately after their preparation (Table S1) and at pre-set times: 7, 30, 90, and 365 days (Figure 2).

### 2.2. Blank Zein Nanoparticles

The size of the blank zein nanoparticles freshly prepared were 294.9 nm for BNp-A and 267.6 nm for BNp-B (Figure 2A). Their pdI values were 0.066 and 0.05, respectively, (Table S1). It is well known [19] that large pdI values correspond to a large size distribution or a nanoparticle aggregation. PdI values below 0.1 will indicate a monodisperse population of nanoparticles. Therefore, BNp-A and BNp-B nanoparticles were found monodispersed and homogenously distributed.

Nanoparticles’ stability was tested at two different temperatures, at mild room temperature (25 °C) and, to mimic storage in a refrigerator, at (4 °C). Blank nanoparticles prepared by method A and stored at 4 °C (BNp-A-F) precipitated after 24 h, while those stored at 25 °C (BNp-A-R) remained unaltered over the studied period (Figure 2A). Blank nanoparticles prepared by method B were found more stable despite the storage temperature. At 25 °C (BNp-B-R), they only precipitated after 90 days (Figure 2A), while no precipitation was observed for the same formulation stored at 4 °C (BNp-B-F). The stability of BNp-B can be attributed to the fact that the particle size does not change over time.

The value of the zeta potential gives an idea of the stability of the nanoparticle dispersion. When the zeta potential is larger than +15 mV or smaller than −15 mV the degree of stability is high [20]. For values of zeta potential within the interval [−15, +15] mV effects of aggregation, coagulation or flocculation appear due to van der Waals interparticle attraction. In our study, both nanoparticles BNp-A and BNp-B showed a zeta potential value higher than +15 mV at both temperatures 25 °C and 4 °C. BNp-B was found stable in all cases. However, BNp-A at 4 °C precipitates. Clearly, temperature determines the nanoparticles’ stability.

### 2.3. LAM-Loaded Zein Nanoparticles

The LAM-loaded zein nanoparticles presented an important difference in size when prepared by method A, 287.0 nm, with respect to method B, 208.8 nm, unlike the blank nanoparticles. They formed nanoparticles with homogeneous distribution (pdI 0.048 and 0.057, respectively) and presented a zeta potential over +30 mV, indicating a greater level of stability. The larger size observed in method A could be explained by the loading of the surfactant in the matrix of the nanoparticles, as it has been mixed together with zein previously to the nanoparticles´ formation and as such, could have occupied more extensively the protein pockets. Contrariwise, the surfactant could have been disposed majorly in the surface when the method B was used, possibly improving the protein packing and nanoparticles dispersion, reducing as a result their size, even when compared to the blank formulation. The LAM-loaded nanoparticles remained mostly stable in terms of size and zeta potential (>+27.4 mV) over 90 days (Figure 2B).

### 2.4. C_3_(LA)_2_-Loaded Zein Nanoparticles

The nanoparticles containing C_3_(LA)_2_ presented slight differences in size when prepared by method A (341.1 nm) and B (308 nm) and were also homogenously distributed, with a pdI of 0.204 and 0.12, respectively (Table S1). These small differences may be due to the residual cationic charge of the arginine guanidino group from the interaction with zein. C_3_(LA)_2_ have a positive charge in each of the arginine guanidino groups, therefore, when coating the zein nanoparticles with the C_3_(LA)_2_ surfactant by method B, repulsions between the cationic charges are produced, preventing aggregation and particle size growth. The storage temperature of the C_3_(LA)_2_ zein nanoparticles did not change the particle size. No precipitation was found in these nanoparticles during this study (Figure 2C).

### 2.5. C_6_(LA)_2_-Loaded Zein Nanoparticles

Unlike LAM and C_3_(LA), the nanoparticles containing C_6_(LA)_2_ presented a slight larger size when prepared by method B (238 nm) compared to method A (200.5 nm). Differences in size between the two methods could be explained by the limited incorporation of the surfactant molecules into the zein molecule when using method A. To form the nanoparticles, the hydrophobic groups of zein pack between themselves forming a domain. The presence of hydrophobic six carbon atoms of the spacer chain of C_6_(LA)_2_ could play an important allosteric blockage, leaving the surfactant mainly on the surface which leads to repulsion between the particles and a decrease in size. They were homogeneously distributed (pdI 0.005) and presented high zeta potential values (>+49.9 mV). Nevertheless, the NpC_6_(LA)_2_-A nanoparticles stored in the refrigerator (NpC_6_(LA)_2_-A-F) (Figure 2D) precipitated after 24 h.

### 2.6. C_9_(LA)_2_-Loaded Zein Nanoparticles

Nanoparticles containing C_9_(LA)_2_ also presented a higher size when prepared by method B (229.1 nm) than method A (180.9 nm) (Figure 2E), which could confirm the hypothesis of non-entrapment of these surfactants in the primary structure of zein. Therefore, the nanoparticles would be formed and the surfactants should be entrapped on the surface of the nanoparticles. This result is reinforced for nanoparticles containing C_9_(LA)_2_ as the zeta potential was approximately the same in both formulations, +50.5 for method A and +49.7 for method B (Figure 2E). This could demonstrate the deposition and stabilization of the nanoparticles´ dispersion of the surfactants. The zeta potential decreased for NpC_9_(LA)_2_-A in both storage conditions at day 30. Zeta-potential of NpC_9_(LA)_2_-B-R showed a great decrease, from +62.2 to +36.3 mV, while for NpC_9_(LA)_2_-B-F stored at 4 °C the z-potential value after 90 days was found to be very stable (Figure 2E).

From the obtained results it can be assessed that the stability of the nanoparticles was reduced in the nanoparticles containing the Gemini surfactants with six, C_6_(LA)_2_ and nine C_9_(LA)_2_ carbon atoms in the spacer chain. Although it was not homogenous, the nanoparticles obtained according to method A were found to have a lower stability at low temperatures. In view of this, a reasonable hypothesis is that the spacer chain plays an important role in the stability of the surfactant within the nanoparticles, which could be due the interaction between those and the amino acid residues of zein [7].

### 2.7. TEM Morphological Appreciation

**Figure 3:**
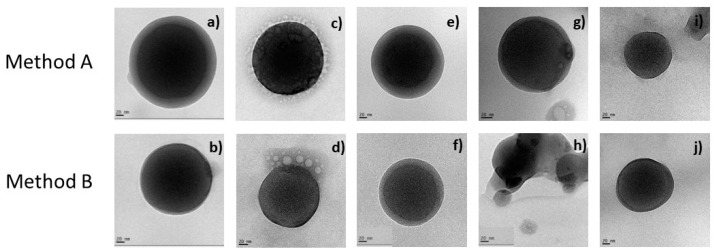
TEM images. Blank zein nanoparticles (**a**) 228 nm and (**b**) 179 nm. LAM/zein nanoparticles (**c**) 141 nm and (**d**) 129 nm. C_3_(LA)_2_/zein nanoparticles (**e**) 194 nm and (**f**) 136 nm. C_6_(LA)_2_/zein nanoparticles (**g**) 214 nm and (**h**) 164 nm. C_9_(LA)_2_/zein nanoparticles (**i**) 132 nm and (**j**) 121 nm. Magnification 40,000×.

The blank and surfactant-loaded zein nanoparticles were observed by transmission electronic microscopy (TEM). The images obtained allowed the identification of the formulation and the evaluation of the morphology and the dispersion of the nanoparticles´ images. The results are shown in Figure 3.

Zein nanoparticles show a spherical, unitary and uniform morphology (Figure 3). The diameters measured in the TEM images of the nanoparticles prepared by method A are slightly higher than those obtained when method B was used (Figure 3). For the nanoparticles of LAM some micelles-like structures were found surrounding the nanoparticles´ structure; while, for the Gemini surfactants, as the length of the spacer chain increases, the diameter of the nanoparticles decreased. These results confirmed the size results obtained by the DLS technique. (Table S1 and Figure 3). The diameter of the nanoparticles containing C_6_(LA)_2_ and C_9_(LA)_2_ (Table S1) have the same value obtained from both methods.

All the nanoparticles obtained have a uniform spherical shape and from the results of the TEM analysis, size and zeta potential measurements, a good stability for nanoparticles containing the surfactant tested was found, despite the preparation method used.

### 2.8. NMR Studies

NMR experiments have been gradually gaining interest in the detection or characterization of macromolecule–ligand or supramolecular–ligand interactions. The STD experiment provides a simpler access to the ligand binding epitope and to other relevant aspects such as the ligand binding affinity in the regime of weak–intermediate affinity (K_D_ between 10 mM to 10^−4^ mM) [21]. The waterLOGSY experiment relies on the transference of NOE from the water resonance. In a sample containing a small molecule and a macromolecular receptor, the appearance in the spectrum of signals of the small molecule with the same phase as the water peak proves that there is weak to intermediate binding affinity among the two molecules and that water is present at the macromolecule binding site [22]. The NOE-exchange relayed (NOEexch) experiment [23] is aimed to detect high-affinity ligands for species with sufficiently long T_1_ relaxing protons (K_D_ between 1 mM up to 10 nM) producing a plot of normalized signal intensity with a characteristic ‘dipping’ curve for some of the ligand protons that is indicative of binding. We used those experiments to evaluate the surfactants: the zein mechanism of interaction and the role of the water as a co-solvent in the complexes formed under the conditions in which the sample is forming a colloidal dispersion, as it is required for the nanoparticles’ formation.

**Figure 4:**
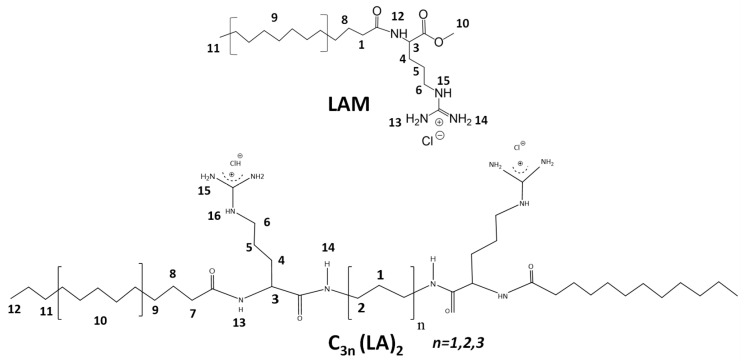

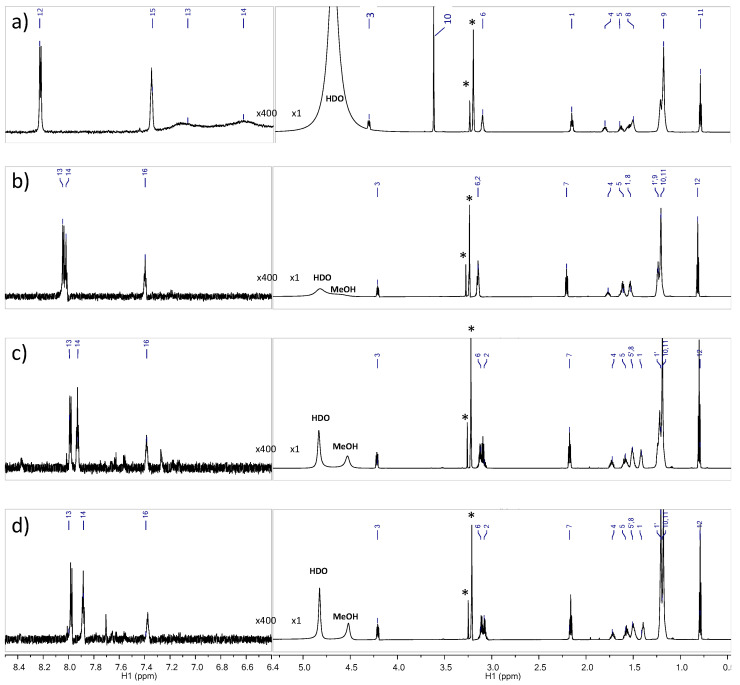
^1^H NMR spectra of pure surfactants (**a**) LAM, (**b**) C_3_(LA)_2_, (**c**) C_6_(LA)_2_ and (**d**) C_9_(LA)_2_ dissolved in H_2_O:CD_3_OD 4:5 *v*/*v* showing the assignment according to the numbering of the chemical structure represented above (Table S2). The left side shows the spectrum in the region from 6.4 to 8.5 ppm with the vertical scale rescaled 400× to show the peaks. Some impurities are denoted with asterisks.

Figure 4 shows the ^1^H NMR spectra correspond to the pure surfactants used to prepare the nanoparticles. LAM is the single chain surfactant counterpart of the arginine Gemini surfactants C_3_(LA)_2_, C_6_(LA)_2_, C_9_(LA)_2_ in which the difference remains solely in the space chain. Therefore, their ^1^H spectra of the four surfactants are very alike, variating mostly in the intensity of the peaks. Table S2 lists the corresponding peaks of each surfactant.

**Figure 5:**
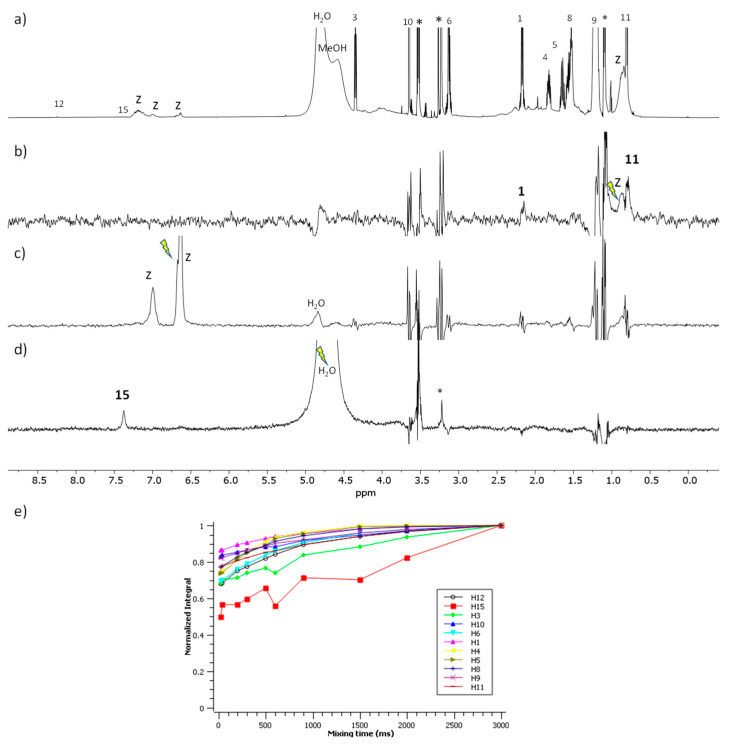
NMR binding interaction study between surfactant LAM and zein. (**a**) ^1^H NMR (**b**,**c**) STD^off-on^ with on-saturation applied over the signal of zein at (**b**) 0.82 ppm and (**c**) at 6.5 ppm (**d**) waterLOGSY spectrum with mixing time 500 ms. In (**b**–**d**) the signal selectively saturated or inverted is indicated with a ray symbol and the assignment of the relevant responses is indicated. Some impurities are denoted with asterisks. The letter z refers to zein signals. (**e**) Plot of the normalized signal intensity of the surfactant protons as a function of mixing time of the NOE-exch spectra.

The spectra corresponding to the mixture of LAM with zein are shown in Figure 5. The STD^off-on^ spectrum (Figure 5b) shows the responses of the protons H-1 and H-11 of the surfactant upon saturation of the aliphatic protons of zein. The waterLOGSY (Figure 5c) shows the response of the proton H-15 of the surfactant which is mediated by bound water molecules. The NOE-exch curves (Figure 5e) did not detect binding given that the normalized intensity essentially grows monotonously for all the protons considered [23]. In summary, these results prove the formation of a complex LAM–zein involving the aliphatic side chain of LAM and the positively charged guanidium group with this latter being in contact to bound water attached to the zein.

**Figure 6:**
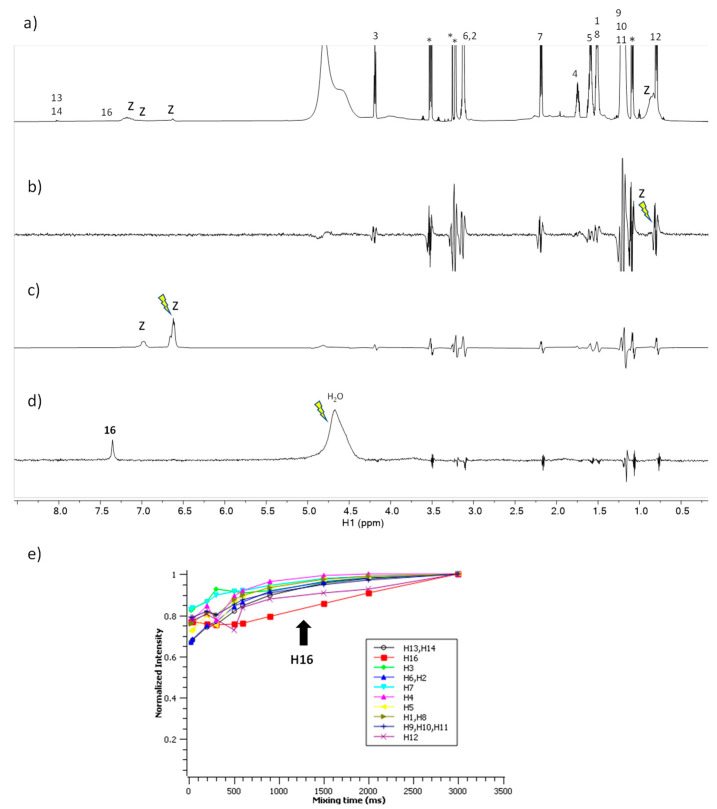
NMR binding interaction study between surfactant C_3_(LA)_2_ and zein. (**a**) ^1^H NMR (**b**,**c**) STD^off-on^ with on-saturation applied over the signal of zein at (**b**) 0.82 ppm and (**c**) at 6.5 ppm (**d**) waterLOGSY spectrum with mixing time 500 ms. In (**b**–**d**) the signal selectively saturated or inverted is indicated with a ray symbol and the assignment of the relevant surfactant responses is indicated. Some impurities are denoted with asterisks. The letter z refers to zein signals. (**e**) Plot of the normalized signal intensity of the surfactant protons as a function of mixing time of the NOE-exch spectra.

The spectra corresponding to the mixture of C_3_(LA)_2_ with zein are shown in Figure 6. The STD^off-on^ spectra (Figure 6b,c) do not show any response to this surfactant upon saturation of the zein protons. In contrast, the waterLOGSY (Figure 6d) shows the response of the proton H-16 to the surfactant mediated by bound water molecules. The NOE-exch curves (Figure 6e) detect a dip for the curve of H-16 of the surfactant which is an indication of binding. In summary, these results prove that the formation of a complex C_3_(LA)_2_ –zein involving the positively charged guanidium group is in contact to bound water that is attached to the zein.

**Figure 7:**
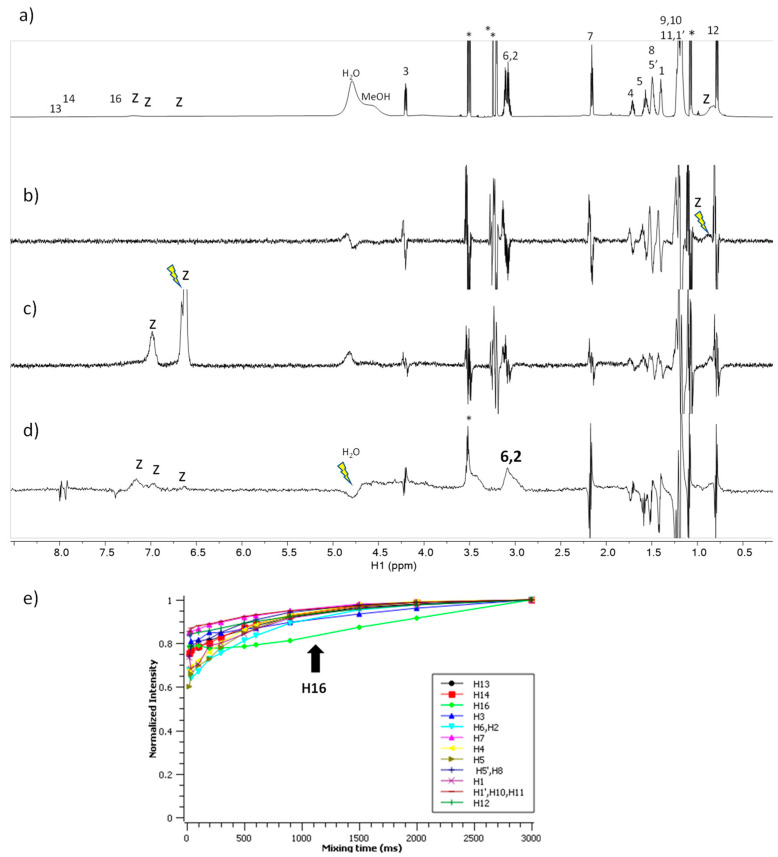
NMR binding interaction study between surfactant C_6_(LA)_2_ and zein. (**a**) ^1^H NMR (**b**,**c**) STD^off-on^ with on-saturation applied over the signal of zein at (**b**) 0.82 ppm and (**c**) at 6.5 ppm (**d**) waterLOGSY spectrum with mixing time 500 ms. In (**b**–**d**) the signal selectively saturated or inverted is indicated with a ray symbol and the assignment of the relevant responses is indicated. Some impurities are denoted with asterisks. The letter z refers to zein signals. (**e**) Plot of the normalized signal intensity of the surfactant protons as a function of mixing time of the NOE-exch spectra.

The spectra corresponding to the mixture of C_6_(LA)_2_ with zein are shown in Figure 7. The STD^off-on^ spectra (Figure 7b,c) do not show any response to this surfactant upon saturation of the zein protons. In contrast, the waterLOGSY (Figure 7d) shows the responses of the protons H-2 and H-6 to the surfactant mediated by bound water molecules. The NOE-exch curves (Figure 7e) detect a dip in the curve of H-16 of the surfactant which is an indication of binding. In summary, these results prove the formation of a complex C_6_(LA)_2_ –zein in which protons H-2 and H-6 are in contact to bound water attached to the zein and that also involves the positively-charged guanidium group.

**Figure 8:**
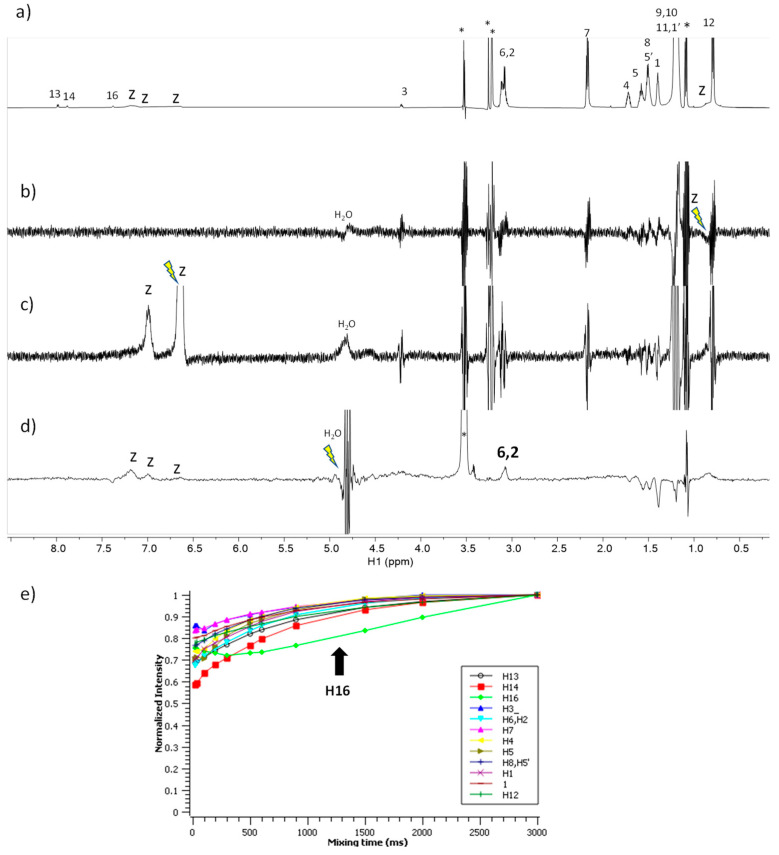
NMR binding interaction study between surfactant C_9_(LA)_2_ and zein. (**a**) ^1^H NMR (**b**,**c**) STD^off-on^ with on-saturation applied over the signal of zein at (**b**) 0.82 ppm and (**c**) at 6.5 ppm (**d**) waterLOGSY spectrum with mixing time 500 ms. In (**b**–**d**) the signal selectively saturated or inverted is indicated with a ray symbol and the assignment of the relevant responses is indicated. Some impurities are denoted with asterisks. The letter z refers to zein signals. (**e**) Plot of the normalized signal intensity of the surfactant protons as a function of mixing time of the NOE-exch spectra.

The spectra corresponding to the mixture of C_9_(LA)_2_ with zein are shown in Figure 8. The STD^off-on^ spectra (Figure 8b,c) do not show any response to this surfactant upon saturation of the zein protons. In contrast, the waterLOGSY (Figure 8d) shows the responses of the protons H-2 and H-6 to the surfactant mediated by bound water molecules. The NOE-exch curves (Figure 8e) detect a dip in the curve of H-16 of the surfactant which is an indication of binding. In summary, these results prove the formation of a complex C_9_(LA)_2_ –zein with very similar characteristics to the aforementioned for C_6_(LA)_2_.

### 2.9. Molecular Docking Studies

**Figure 9:**
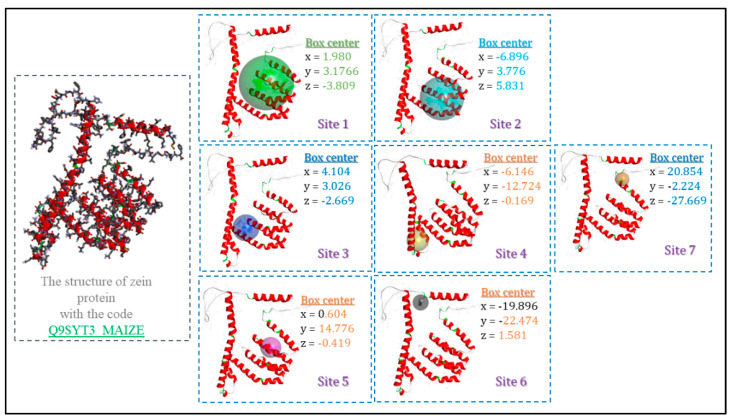
Different binding sites and their coordinates (x, y and z) on the surface of the zein structures (Q9SYT3_MAIZE) using Discovery Studio Client v16 software.

The molecular docking results give us a rough idea of how the ligand (surfactants) and the receptor (zein) complexes are brought into contact to form the nanoparticles. We have scanned all the possible active sites on the surface of the macromolecular zein structure using Discovery Studio Client v16 software. Seven binding site possibilities were detected (Figure 9).

The synthesized compounds showed good binding energy values in the different binding sites. From the Tables S3–S5, it was observed that all the compounds exhibited high free energy binding between −4 and −9 kcal/mol.

**Figure 10:**
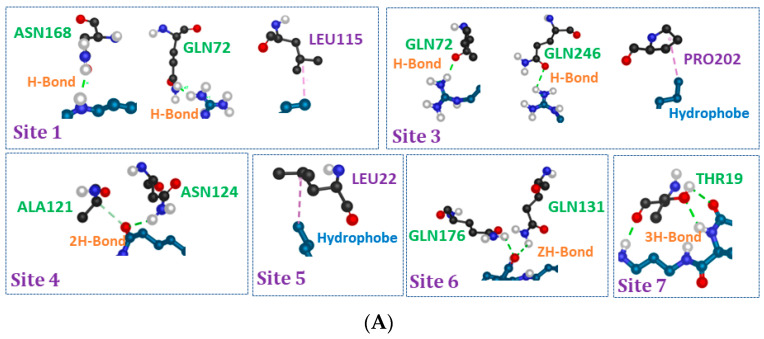

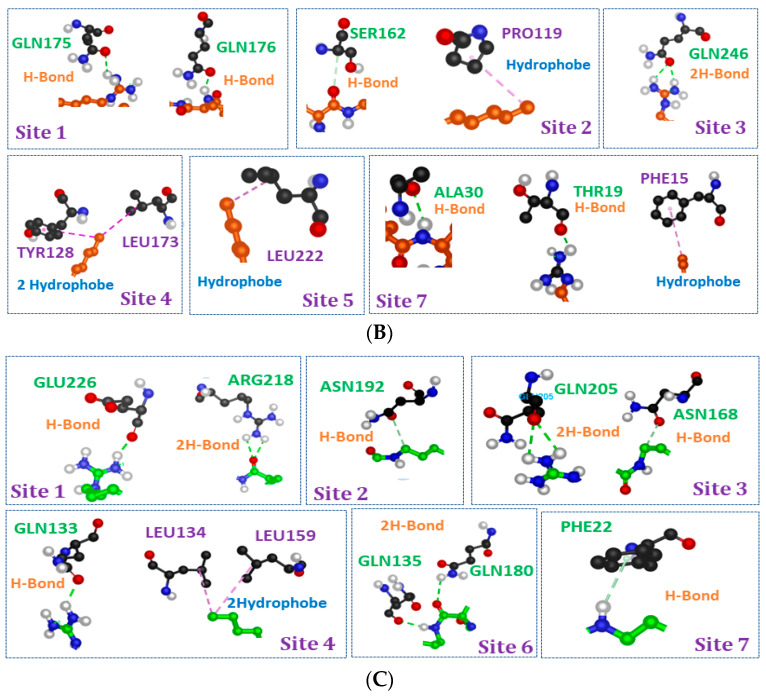
Different types of interaction on the surface of the zein structures (Q9SYT3_MAIZE) in different binding sites for (**A**): C_3_(LA)_2_ (**B**): C_6_(LA)_2_ and (**C**): C_9_(LA)_2_.

All the investigated analog ligands showed promising good interactions with different amino acid residues (Figure 10). It should be noted that in the active sites 2, 6 and 5 for C_3_ (LA)_2_, C_6_ (LA)_2_ and C_9_ (LA)_2_, respectively, no interaction was detected, while in the other active sites, a different number of hydrogen and hydrophobic interactions against different residues were found.

**Figure 11:**
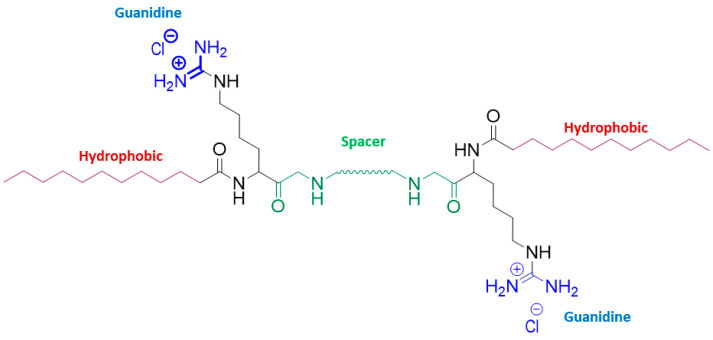
Different parts of the molecular structure of surfactants that interact with zein structures (Q9SYT3_MAIZE).

According to the modes of interaction, the core parts in the molecular structure of the surfactants which interact with the structure of the protein are as shown in Figure 11: the two parts hydrophobic, hydrophilic and spacer chain.

Most of the interactions observed in the different active sites for the three ligands are driven by carbonyl and amide groups belonging to the spacer moiety. Sometimes the polar and hydrophobic parts present interactions in some active sites on the surface of zein protein. Among the sites, the contribution of the polar heads in the interactions was noticed solely in two sites for C_3_ (LA)_2_ and three sites for both C_6_ (LA)_2_ and C_9_ (LA)_2_. Bearing in mind that the molecular structure of these surfactants contains two polar heads, and only one participates when the hydrophilic part is enrolled in the interactions to zein (Figure 11), one is always free, which also justifies the positive zeta potential found in the nanoparticles. The same remark was also noted for the hydrophobic parts. When the alkyl parts participate in the interaction, one chain interacts, while the other chain is always free and does not take part in the interaction (Figure 11).

### 2.10. Surface Pressure/Area Isotherms

#### 2.10.1. Single Component Systems

The Langmuir trough–Wilhelmy plate method has been widely used to measure the surface pressure-area isotherms of monolayers spread at air–water interfaces. The method is based on spreading the monolayer at the air–water interface and then compressing it. The trough measures the increase in the surface pressure, π, defined as the difference between the surface tension measured for pure water, γ_0_, and the surface tension measured for the monolayer, γ.

In this paper, surface pressure-area measurements were performed to investigate the adsorption at the water/air interface of the cationic Gemini arginine surfactants and also their mixtures with zein.

**Figure 12:**
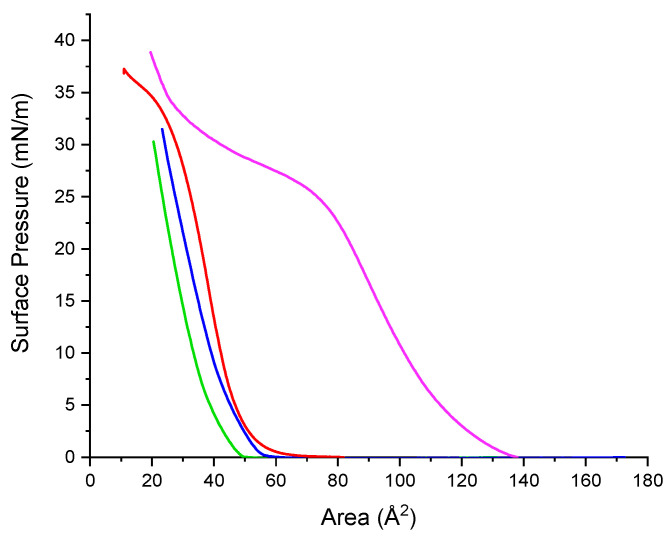
Surface pressure isotherms of zein and arginine-based Gemini surfactants. Zein (red line), C_3_(LA)_2_ (blue line), C_6_(LA)_2_ (green line), C_9_(LA)_2_ (purple line). For Gemini the area corresponds to molecular area, for zein the area corresponds to the area per amino acid residue.

Although basically all the studies on surface pressure-area isotherms involve amphiphiles that are practically insoluble in the subphase, soluble surfactants monolayers can also be compressed in some cases. For example, Gemini arginine surfactants are partially soluble in water. Hence, they do not form high stable monolayers against surface pressure and their isotherms show a characteristic profile for partially soluble surfactants. Results are shown in Figure 12.

Under compression, monolayers of the Gemini arginine surfactants form expanded gas and liquid phases. Their isotherms show that the larger the spacer chain, the more favorable is the adsorption of the surfactant at the air–water interphase. For C_9_(LA)_2,_ the surface pressure values are observed over wider areas than those of both C_3_(LA)_2_ and C_6_(LA)_2_ that show isotherm shapes that rapidly grow within a narrow area region. Gemini C_9_(LA)_2_ form more stable monolayers than their homologues, as suggested by the flat collapse shown in Figure 12.

In addition to the solubility, the differences observed on the monolayer can be attributed to the arrangement of the molecules of the cationic Gemini surfactants at the air–water interface. Short spacer chains (C_3_, C_6_) entails reduced head group separation, leading to a head group with a high charge density and therefore the presence of repulsion forces between them which destabilizes the monolayer [24,25]. For longer spacer chains (C_9_) [26,27,28] as the monolayer is progressively compressed the spacer chain penetrates into the two hydrophobic chains forming part of the hydrophobic component of the surfactant and thus improving the cohesion forces between the molecules and the stability of the monolayer.

Figure 12 shows plots of the surface pressure-area isotherm of zein at the water/air interface. The isotherm indicates that zein forms stable monolayers starting at a low surface pressure in the expanded state, followed by a steep increase in surface pressure as liquid condensate. At a pressure of 30 mN/m, which corresponds to about 27 Å^2/^residue, the beginning of a transition is observed.

#### 2.10.2. Mixed Systems

**Figure 13:**
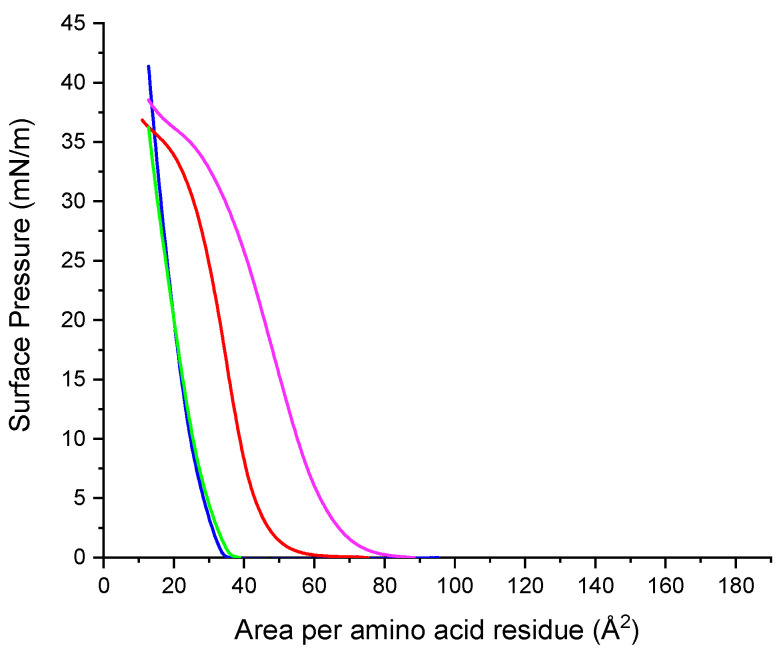
Isotherms of zein/Gemini arginine surfactants. zein (red line), zein/C_3_(LA)_2_ mixture (blue line), zein/C_6_(LA)_2_ mixture (green line), zein/C_9_(LA)_2_ mixture (purple line).

The compression isotherms of arginine surfactants zein-systems were studied to know the extent to which the mixture compounds are forced into the bulk subphase. If the mixed isotherm is similar to that of the pure zein, it can be suggested that the surfactant desorbed totally when the mixed monolayer was compressed and weak or no interactions were present. Thus, any deviation from the isotherm of a pure zein monolayer can be related to the incomplete desorption of the arginine surfactants caused by the presence of interactions between monolayer components. Figure 13 shows the isotherms of arginine surfactant–zein mixtures. The profile of the obtained isotherms showed significant changes (Figure 13) compared to the isotherm of zein alone (Figure 12).

The isotherm of the C_9_(LA)_2_–zein mixture is shifted to larger molecular areas. When it is compared to the profiles of each component of the mixture (Figure 12), it is observed that in the mixture the flat collapse observed in the C_9_(LA)_2_ isotherm has disappeared and the corresponding profile is similar to that of zein alone but moved to higher molecular areas. This shift suggests some kind of interaction between the two components of the mixture related to occupied area.

The profile isotherms of C_3_(LA)_2_ mixtures with zein are similar to that of C_6_ (LA)_2_ and both are displaced to lower molecular areas with respect to the profile of zein alone (Figure 13). This displacement could be interpreted as a dissolution towards the subphase of the components of the monolayer due to the formation of soluble mixed aggregates.

As the three studied surfactants have two cationic charges in the arginine guanidino groups, in theory the ionic interactions of these groups with zein should be the same for the three surfactants, so the observed differences of the isotherm profile of the C_9_ (LA)_2_–zein mixture should have their origin in the hydrophobic interactions. In the air/water interface, the zein molecules oriented their polar residues to water subphase and the nonpolar residues to the air [29]. In this scenario, as C_9_(LA)_2_ has a large spacer chain which may form part of the hydrophobic, it can interact with the zein hydrophobic residues, reinforcing the zein–surfactant interactions and increasing the area occupied by the two components of the monolayer.

### 2.11. Mechanical properties

The mechanical properties of the monolayer can be inferred from its compressibility modulus [30]. The compressibility modulus (C) of the monolayer was computed from the surface pressure, π and the area, A, data according to the equation

C = −1/A (δA/δπ)

and the elastic modulus (E) that measures the resistance to elastic deformation is defined as

E = 1/C = −A (δπ/δA)

To gain further insight into the surface properties of the Gemini arginine–zein monolayers we analyzed the above compression isotherms (Figure 13) in terms of the E modulus as a function of π. In general, the value of E depends on the state of the monolayer [31], in such a way that high values of E appear when the rigidity of the monolayer is high due to strong interfacial cohesive forces. For instance, high values of E (100–200) are typical of condensed phases.

**Figure 14:**
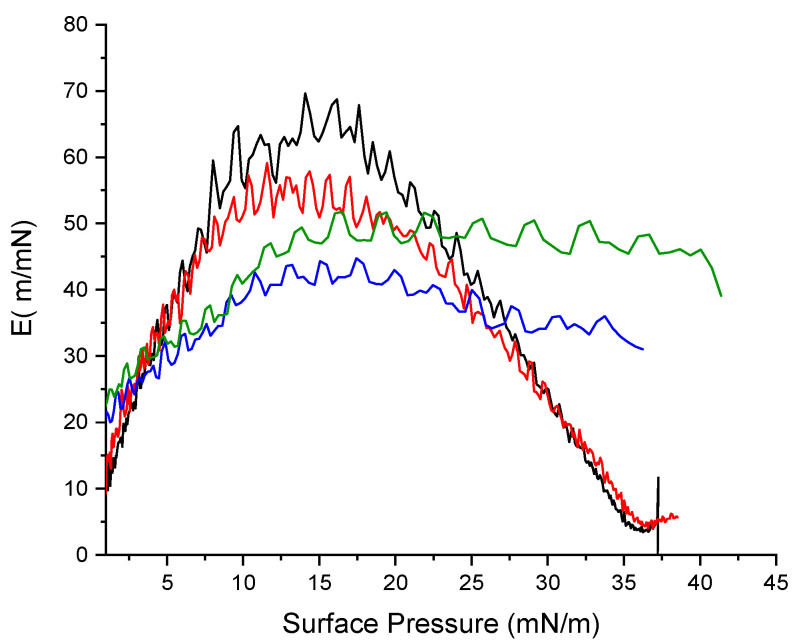
Elastic modulus as a function of surface pressure. Zein (red line), C_3_(LA)_2_ (blue line), C_6_(LA)_2_ (green line) and C_9_(LA)_2_ (black line).

Plots of E as a function of π are shown in Figure 14. The values of E for zein increased with the increment of the surface pressure, reaching a maximum close to 70 m/mN, which corresponds to the almost condensed liquid phase. The shapes of the C_9_(LA)_2_–zein mixture and zein are parabolas that share their starting and ending segments but with π zein values being always higher. This aspect indicates that C_9_(LA)_2_ interacts favorably with zein in such a way that it does not destabilize the monolayer. Plots of the rest of the surfactants present a different profile with maximum values of E being lower than those of the C_9_(LA)_2_–zein mixture and zein alone. These results indicate that the monolayers of these mixtures, under compression, could have been partially solubilized.

### 2.12. Antimicrobial Activity

**Figure 15:**
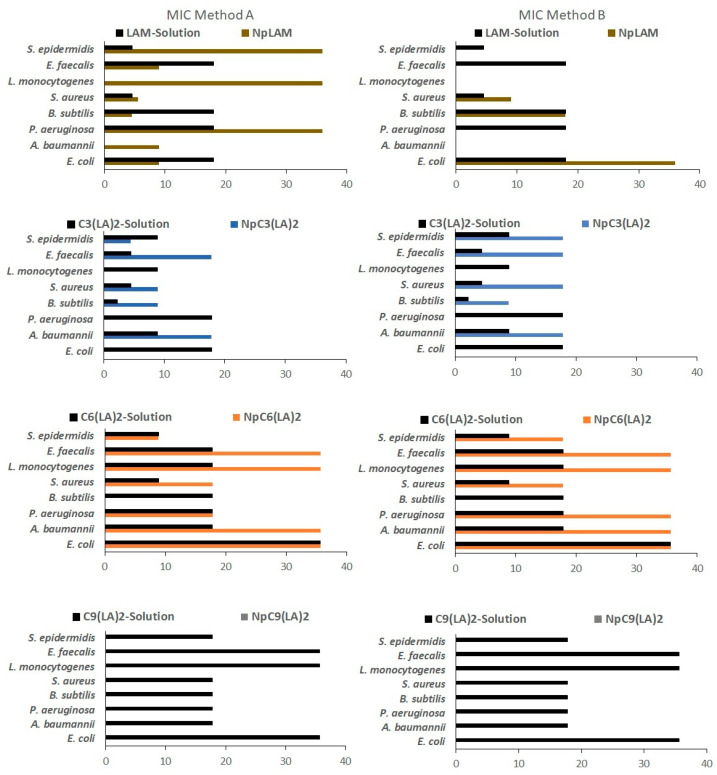
Determination of the MIC (µg/mL) for LAM, C_3_(LA)_2_, C_6_(LA)_2_, C_9_(LA)_2_ solutions and loaded zein nanoparticles prepared by methods A and B against bacteria.

**Figure 16:**
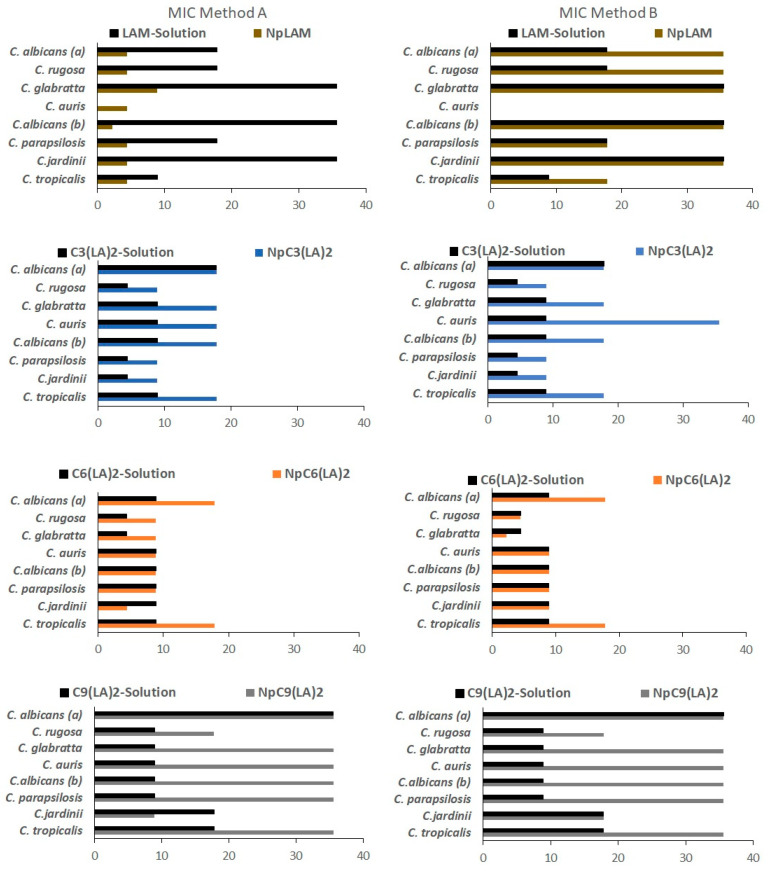
Determination of the MIC (µg/mL) for LAM, C_3_(LA)_2_, C_6_(LA)_2_, C_9_(LA)_2_ solutions and loaded zein nanoparticles prepared by methods A and B against yeasts. *Candida albicans* ATCC 10,231 (a) and *Candida albicans* ATCC 90,028 (b).

The increasing resistance of microorganisms to conventional antimicrobial agents demands an urgent search for new alternatives. Resistance is noticeable, for example, in the treatment of nosocomial infections, which are expensive and (not always) efficient. Cationic surfactants have become potential candidates to overcome this problem; besides their noteworthy antimicrobial activity, their mechanism of action limits the development of resistance in sensible microorganisms [1,2]. Here, MIC, MBC/MFC values for the bacteria and the yeasts were determined for the blank zein nanoparticles, BNp-A and BNp-B, and loaded surfactant-zein nanoparticles prepared by methods A and B (NpLAM-A and B, NpC_3_(LA)_2_-A and B, NpC_6_(LA)_2_-A and B, NpC_9_(LA)_2_-A and B). In addition, as a control, MIC and MBC/MFC were determined for plain surfactant solutions at 35.6 µg/mL, which is the same concentration loaded in the nanoparticles. The results are shown in Figure 15 and Figure 16.

The antibacterial activity of LAM nanoparticles is displayed in Figure 14. LAM nanoparticles prepared by method A (NpLAM-A) presented very low MIC values for most bacteria tested, and in some cases exhibited better antimicrobial activity than the free surfactant. However, when method B was used (Np-LAM-B) the nanoparticles showed higher MIC values than LAM in the solution for all strains tested. No MBC were found against *L. monocytogenes* for LAM nanoparticles, despite the preparation method (Figure S1) and the same was observed for NpLAM-B and against *A. baumannii* (Figure S1). In contrast, NpLAM-A showed a microbicidal activity against both bacteria (Figure S1), unlike the pure surfactant solution which was not active.

C_3_(LA)_2_ solution exhibited higher antimicrobial activity than LAM. In general, Gemini surfactants show better antimicrobial efficiency than their single counterparts. For arginine Gemini surfactants the combination of their structural features (two positive charges and two alkyl chains per molecule) and their physicochemical properties (low critical micellar concentration and high solubility) seems to improve the electrostatic and hydrophobic interactions of these surfactants with the bacterial membranes [1].

C_3_(LA)_2_ nanoparticles (NpC_3_(LA)_2_) showed lower activity than the free surfactant although they maintained a very potent activity with MIC values lower than 20 µg/mL. These nanoparticles did not present inhibitory activity against *P. aeruginosa*, *E. coli* and *L. monocytogenes* (Figure 15). Regarding their bactericidal activity (MBC), it was found that these aggregates can be considered good bactericidal agents against *S. aureus* and *E. faecalis* (MBC 17.8 µg/mL), while no bactericide activity was observed for the other strains (Figure S1).

C_6_(LA)_2_ nanoparticles (Np C_6_(LA)_2_) showed similar or lower activity than pure solution of this Gemini surfactant. Regarding the preparation method, the nanoparticles Np C_6_(LA)_2_-A and Np C_6_(LA)_2_-B presented similar MIC values against *P. aeruginosa*, *E. coli* and *E. faecalis*, while none of them had any effect over *B. subtilis* (Figure 15). The bactericidal effect of NpC_6_(LA)_2_-A against *A. baumannii* and *P. aeruginosa* was similar. Moreover, NpC_6_(LA)_2_-B and pure C_6_(LA)_2_ solution also showed similar MBC values against *S. epidermidis* (Figure S1).

C_9_(LA)_2_ nanoparticles (NpC_9_(LA)_2_) (Figure 15) did not present inhibitory nor bactericide activity against the bacteria tested (Figure S1), unlike its solution that did show inhibitory and bactericide activity against most strains tested, with the exception of *B. subtilis* (Figure 15 and Figure S1).

Figure 16 and Figure S2 show the activity of these systems against representative yeasts. Np-LAM-A exhibited very low MIC values against all microorganisms, it was noteworthy active (MIC 4.45 μg/mL) against *C. auris*, a novel *Candida* species that has been rapidly spreading and has shown high resistance to conventional antifungal drugs. The encapsulation of LAM in these nanoparticles using the method A improved the activity of this surfactant against the yeasts tested. However, nanoparticles prepared using method B were found to have lower activity than LAM in solution. The MFC followed the same tendency as the MIC values found for LAM nanoparticles prepared by methods A and B, where an equal or improved activity was found against most yeast, except for *C. auris*, *C. paropsilosis* and *C. tropicalis* (Figure 16 and Figure S2).

The Gemini C_3_(LA)_2_ pure solution showed better antifungal activity than its single chain homologue LAM. The C_3_(LA)_2_ nanoparticles (Np C_3_(LA)_2_) exhibited good antifungal efficiency (MIC values lower than 20 μg/mL) although the activity of the surfactant slightly decreases when it is incorporated into zein the nanoparticles and their efficiency was not affected by the preparation method (Figure 16 and Figure S2).

C_6_(LA)_2_ nanoparticles (NpC_6_(LA)_2_) also presented very good activity with a similar inhibitory activity to its solution against the yeasts tested (Figure 16), except for *C. tropicalis* and *C. albicans*, in which the MIC values of the nanoparticles were higher. The activity of the NpC_6_(LA)_2_ was in general lower than that obtained with the NpC_3_(LA)_2._ Notice than six of the eight strains tested exhibited MIC values lower than 10 µg/mL while for the other two strains the MIC values were lower than 20 µg/mL (Figure 16). The MFC was partially equivalent to the C_3_(LA)_2_ solution (Figure S2).

Unlike the other Gemini surfactants, C_9_(LA)_2_ nanoparticles (NpC_9_(LA)_2_) exhibited remarkably higher MIC values against the yeasts tested, compared to it solution (Figure 16). Even so these nanoparticles also retain the antifungal activity with MIC values lower than 40 μg/mL (Figure 16). The MFC for NpC_9_(LA)_2_-A and specially NpC_9_(LA)_2_-B was also higher (Figure S2). A similar activity for both nanoparticles and its solution was maintained solely against *C. jadinni* and *C. albicans*.

It has been reported that smaller nanoparticles have larger specific surface areas which result in better antimicrobial systems because these aggregates have greater probability to pass through the bacterial cell membrane [32]. Moreover, high positive zeta potential values improve the electrostatic attraction between positively-charged nanoparticles with the negatively-charged cell membranes, resulting in a higher inhibition of bacterial growth. In this case, the NpC_9_(LA)_2_ nanoparticles were smaller than NpC_3_(LA)_2_ and NpC_6_(LA)_2_; moreover, their zeta potential values were also higher than that of nanoparticles prepared with the other surfactants. This means that, in this case, the size and the zeta potential are not the main factors that control the antimicrobial activity of these systems; some other physicochemical properties such as shape, surface morphology and crystal structure can also affect the antibacterial effectivity of these formulations. These behaviors have already been reported for other systems; for Mg(OH)_2_ nanoparticles, it was found that the smallest aggregates had the weakest antibacterial effect [33].

Regarding the antimicrobial responses, a greater activity was found against yeasts than bacteria, by comparing both the MIC (Figure 15 and Figure 16) and MBC/MFC results (Figures S1 and S2). A discrete decrease in the antimicrobial activity of these amino acid based surfactants was found when nanoencapsulated in the zein nanoparticles; but, in general, these aggregates still maintain a very potent antimicrobial activity. This difference is more notable in the Gemini surfactant with large spacer chains, C_9_(LA)_2_. In fact, the NpC_9_(LA)_2_- nanoparticles were not active against any bacteria tested unlike its solution, which presented still a good activity. The same behavior has been observed when phenylalanine–arginine surfactants were loaded to the zein nanoparticles [34].

It was observed that in most cases the nanoparticles obtained by method A, where the surfactants were dissolved together with zein prior to the nanoparticles´ formation, were more effective than those obtained by method B, in which the surfactants were disposed in the zein nanoparticles previously formed, for both the yeasts and the bacteria. Few cases of low/lack of activity of the surfactant-nanoparticles were observed. The development of antibacterial nanoparticles is a promising approach for combating drug resistance [34]. Most of the antibiotic resistance mechanisms are irrelevant for antibacterial nanoparticles because the mode of action of these aggregates mainly involves direct contact with the bacterial cell wall and its disruption. This mode of action reduces the probability of bacterial drug resistance because the bacterial membrane is difficult to change through only a few genetic mutations [32].

### 2.13. Hemolytic Activity

**Figure 17:**
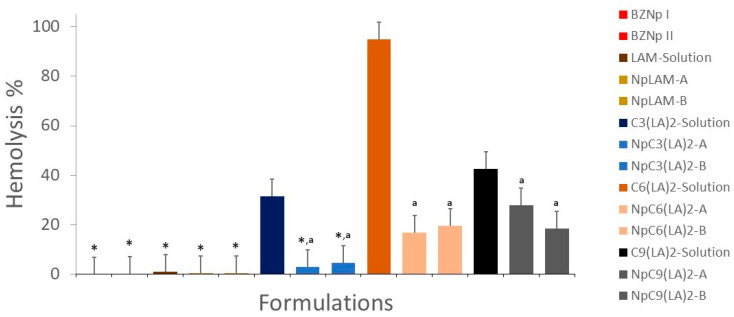
Hemolytic activity of the surfactants´ solutions (35.6 µg/mL) and loaded zein nanoparticles prepared by methods A and B. The results are expressed as a percentage using PBS (pH7.4) (negative control) as reference. * Significatively reduced the hemolytic activity compared to the respective solution (*p* < 0.05). ^a^ Equal to the negative control (*p* > 0.05). Data submitted in pairs to one-way ANOVA with post-hoc Tukey test when *p* < 0.05.

In order to evaluate the impact of the nanoencapsulation of the surfactants over the cellular membranes, the erythrocyte hemolytic activity of the surfactants’ nanoparticles and solutions was evaluated in vitro. The hemolytic activity expressed as a % of hemolysis is summarized in Figure 17.

The encapsulation of the surfactants in the zein nanoparticles reduced drastically the hemolytic activity. Among the surfactants, LAM was found to produce a very low level of hemolysis in solution. Therefore, their nanoparticles (NpLAM-A and NpLAM-B) also maintained their reduced effect (<0.5% hemolysis) (Figure 17).

In contrast, the Gemini surfactants presented a high hemolytic effect in the solution, while the nanoencapsulation was found to reduce this effect. C_3_(LA)_2_, presenting a moderate hemolytic activity in the solution (31.5%), markedly reducing to just 3% and 4.6% for the nanoparticles NpC_3_(LA)_2_-A and NpC_3_(LA)_2_-B, respectively (Figure 17). The C_6_(LA)_2_ presented the highest reduction amongst the Gemini surfactants. Its solution ensued a very high level of hemolysis (94.91%), while the nanoparticles NpC_6_(LA)_2_-A and NpC_6_(LA)_2_-B lead to a substantial decrease on the hemolytic activity to 17.8% and 20.7%, respectively (Figure 17). Therefore, a substantial reduction of approximately 75–80% was obtained after the nanoencapsulation of this surfactant in the zein nanoparticles. In the same way, the C_9_(LA)_2_ solution showed an intermediate hemolytic activity, c.a. 42.6%, and showed a consistent reduction in its hemolytic capacity. The hemolysis was reduced in the nanoparticles NpC_9_(LA)_2_-A and NpC_9_(LA)_2_-B to 28% and 18.6%, respectively.

The drastic reduction in the hemolytic behavior of these surfactants when incorporated in the zein nanoparticles is an extraordinary result. This reduction in the hemolytic activity for the nanoparticles can be due to the interactions found between the hydrophobic residues of the zein and the hydrophobic regions of the surfactant, which would make the surfactant more internalized or immobilized. This internalization would induce the reduction of both the antimicrobial properties and the hemolytic activity [34]. This behavior can be also related to the morphology and size of the aggregates formed by every compound. Aqueous solutions of pure LAM, C_3_(LA)_2_ and C_6_(LA)_2_ contain spherical micelles, then when these compounds are incorporated in the zein nanoparticles the size of the aggregates increases a lot and the hemolysis diminishes substantially. C_9_(LA)_2_ forms viscous solutions with big aggregates such us worm-like micelles [35]. In this case, when this compound was incorporated into the zein nanoparticles the increase of the aggregate’s size was not so marked and the same happened with the hemolytic activity. Our results suggest that the aggregate size and morphology play an important role in the hemolytic character of these formulations. It has been reported that these physicochemical properties also affect significantly the hemolysis of aggregates formed by these Gemini surfactants with cholesterol and dilauroyl phosphatidyl choline (DLPC) [15].

It is noteworthy that the reduction in the hemolytic activity is considerably higher than that observed for the antimicrobial activity. The formation of complexes C_6_(LA)_2_ and C_9_(LA)_2_–zein could explain the reduction in their hemolytic activity when nanoencapsulated in the zein nanoparticles. As the aliphatic moieties were complexed to the protein, the cationic moieties (arginine groups) were still found available to interact biologically with the bacteria and the fungi, maintaining their biocide activity equal to their solution. Nonetheless, the shorter space chain found in C_3_(LA)_2_ made it more hydrophilic, and similarly to LAM, it was not found to cause membrane disruption in the erythrocytes.

According to the docking results, the formation of the zein nanoparticles containing the surfactants is likely to be manifested by interactions in which different groups in the molecular structure of the surfactants are involved. The two polar heads and hydrophobic parts are unlikely involved in the modes of interaction. In three or two active sites, we have eventually seen that one of the polar heads or one of the alkyl chains participates in the interaction mode which indicates that even if the surfactant molecules are engaged in the nanoparticles´ formation, they keep their hydrophobic and electrophilic characteristics. This aspect justifies the antimicrobial power which remains active for the nanoparticles containing the surfactant molecules compared to their free forms.

Notice that biomedical applications of antimicrobial formulations will be important only if they are lethal to bacteria or fungi at concentrations that are not toxic toward mammalian cells. Then it could be assessed that the preparation of these Gemini surfactants-based nanoparticles increases the safety of these antimicrobials and then widens the range of their potential biomedical applications.

## 3. Materials and Methods

### 3.1. Materials

Zein, RPMI 1640 and Resazurin were purchased from Sigma-Aldrich (St. Louis, MO, USA). NaCl, KCl, Na_2_HPO_4_, KH_2_PO_4_ were purchased from Fluka (Fluka Chemie, Buchs, NC, USA); Sabouraud agar, SBA, was purchased from Oxoid (Oxoid Ltd., Basingstoke, Hants, UK); Müller-Hinton broth, MHB, and Müller-Hinton agar, MHA, were from Merck (Merk KGaA, Darmstadt, Germany). 

Four cationic arginine-based surfactants were selected for this study: one single chain, LAM, and three Gemini, C_3_(LA)_2_, C_6_(LA)_2_ and C_9_(LA)_2_ (Figure 1). The single chain N^α^-lauroyl arginine methyl ester (LAM) has one arginine as polar head linked to one fatty chain of twelve carbon atoms. The Gemini contain two symmetrical chains of N^α^-lauroyl-arginine residues linked by amide covalent bonds to an α,ω-alkylidendiamine spacer chain. Structurally, the three Gemini surfactants differ by the length of the spacer chain (*S* in Figure 1), which are 3, 6 and 9 carbon atoms, respectively. The surfactants were synthetized following the protocol described in the study of Perez et al. (1996) [36]. Those molecules were purified by preparative liquid chromatography with a Waters HPLC system equipped with a Kromasil 100 C8 5 µm 25 × 2.12 column (Bohus, Sweden) and characterized by ^1^H NMR. All the other reagents were pure grade and used as received.

### 3.2. Nanoparticles Preparation

**Figure 18:**
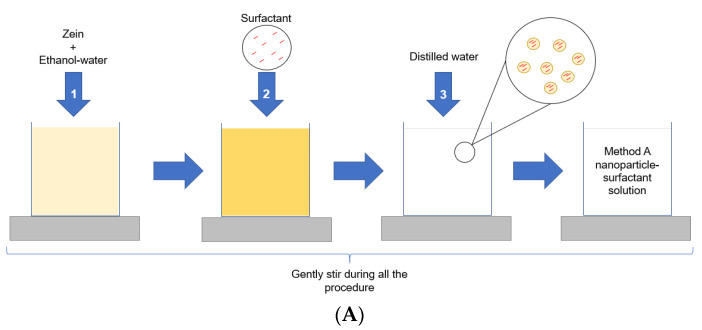

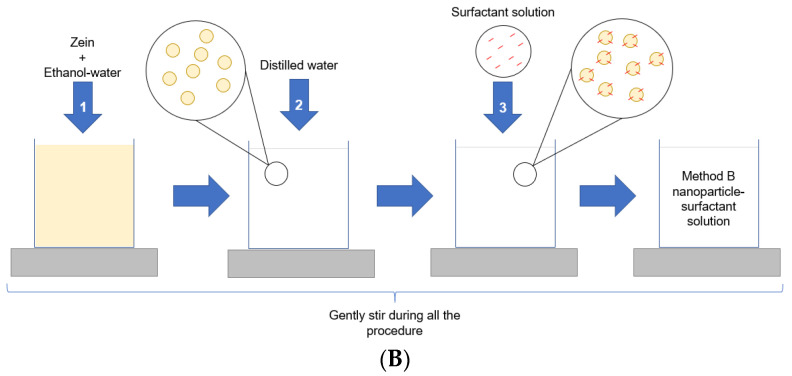
Methods used for the preparation of zein nanoparticles containing the arginine surfactants: Method (**A**)—zein and surfactants are pre-mixed before the nanoparticles´ formation. Method (**B**)—the surfactants are added to pre-formed zein nanoparticles.

Two different protocols were used for the nanoparticles´ preparation at room temperature [35]. The composition of the surfactant-loaded zein nanoparticles is displayed in Table S1, while the preparation methods are illustrated in Figure 18.

The first protocol was designed to promote the incorporation of the surfactant within the nanoparticles´ matrix (Method A) by dissolving Zein and surfactant together before the nanoparticles´ formation (Figure 18A). Briefly, a 0.0712% *w*/*v* zein solution was prepared in 70% *v*/*v* ethanol under constant stirring. After that, 0.0712 g of each surfactant dissolved in 10 mL of ethanol (70%) was incorporated directly to the zein solution at proportion zein/surfactant mass ratio of 1/10 *w*/*w*. Finally, ultrapure water was gradually added to a total volume of 50 mL to induce the nanoparticles formation by nanoprecipitation.

The second protocol aimed to load the surfactant onto the nanoparticles’ surface (Method B) by using pre-formed zein nanoparticles (Figure 18B) [12,18]. Briefly, a 7.91% (*w*/*v*) 0.712 g of zein was dissolved in 9 mL of ethanol (70%) under constant stirring. Next, 40 mL of ultrapure water (Milli-Q system, Micropore^®^, Redcar, UK) was gradually added to promote the nanoparticles’ formation. Separately, 0.0712 g of each surfactant was dissolved in 1 mL of ethanol (70%) and added dropwise to the pre-formed zein nanoparticles, under constant stirring. The final zein/surfactant mass ratio was kept at 1/10 *w*/*w*.

Blank zein nanoparticles (BNp) were prepared in the same manner, except for the surfactants’ loading, and used as controls.

### 3.3. Nanoparticles Characterization and Stability

The nanoparticles were characterized in terms of size, polydispersity index (pdI) and zeta potential (Zetasizer Nano ZS90, Malvern instruments, Malvern, UK). In order to access the best storage conditions, the stability of the formulations was assessed over 365 days under two different conditions in controlled temperature chambers: room temperature (25 ± 2 °C) and low temperature (5 ± 3 °C), according to the ICH guidelines [37,38]. The measurements were performed at 25 °C after 0, 7, 30, 90 and 365 days in triplicate for each sample. The morphology of the nanoparticles was observed with Transmission Electronic Microscopy (JEOL JEM-2010, Electron Microscope, Akishima, Tokyo, Japan). For that, a drop of each dispersion was deposited on a copper grid covered by an amorphous carbon film, dried and submitted for observation.

### 3.4. NMR Spectroscopy

NMR experiments were conducted at 25 °C on a Bruker NEO 17.6 T spectrometer (proton resonance 750 MHz), equipped with a ^1^H-^19^F/^13^C/^15^N triple resonance PA-TXI probe with deuterium lock channel and shielded PFG z-gradient. The spectrometer control software was TopSpin 4.0. The chemical shifts reported are referenced to the lock deuterium solvent. Spectra were processed and analyzed with Mestrenova software v14.0 (Mestrelab Inc., Santiago de Compostela, Spain).

In order to investigate the chemical interactions between the surfactants and zein, the single compounds LAM, C_3_(LA)_2_, C_6_(LA)_2_ and C_9_(LA)_2_ and their binary mixtures with zein were dissolved and homogenized in 0.6 mL of CD_3_OD:D_2_O 9:1 (*v*/*v*). The concentration of zein was fixed at 8 mM, while the mixtures were prepared at a molar ratio surfactant: zein of 50:1. NMR spectra were measured following our previous studies [18,29]. One-dimensional ^1^H spectra were measured for the individual components and for the mixture surfactant–zein. The ^1^H-STD-NMR spectrum was measured for the mixture. The saturation time was set to 2 s and the STD^off^ saturation was applied at 20 ppm. The STD^on^ saturation was applied at 2.0 ppm for surfactant–zein mixture corresponding to a region of the spectrum where proton signals of zein but not surfactant are expected. The STD^on^ and STD^off^ subspectra were measured in alternate scans and subtracted by phase cycling providing the STD^off-on^ spectrum.

NOE-exchange relayed (NOEexch) experiments [23] were acquired for the aforementioned mixtures LAM, C_3_(LA)_2_, C_6_(LA)_2_ and C_9_(LA)_2_ and their binary mixtures with zein dissolved and homogenized in 0.6 mL of CD_3_OD:D_2_O 9:1 (*v*/*v*). The version of the experiment used introduces a T_2_ relaxation filter prior and after the NOESY mixing time, each one with a duration of 40 ms. The experiment was repeated at the following mixing times: 0.02, 0.04, 0.2, 0.3, 0.5, 0.6, 0.9, 1.5, 2.0 and 3 s. Each spectrum was acquired with 128 scans and a total duration of each scan of 6.0 s.

WaterLOGSY experiments [29] were acquired for the individual mixtures of the surfactants (LAM, C_3_(LA)_2_, C_6_(LA)_2_ and C_9_(LA)_2_) and zein dissolved in H_2_O:CD_3_OD 4:5 *v*/*v*. The experiments were performed with a selective 180 degrees’ inversion pulse applied over the water signal at 4.7 ppm by means of a gaussian shaped selective pulse of 7.5 ms covering a band width of 118 Hz (0.15 ppm in our spectrometer). The experiment was repeated with a mixing time of 50 and 500 ms for comparison, while the last was used for plotting the results. Each waterLOGSY spectrum was acquired with 64 scans and a total duration of each scan of 5 s.

### 3.5. Molecular Docking Studies

**Figure 19:**
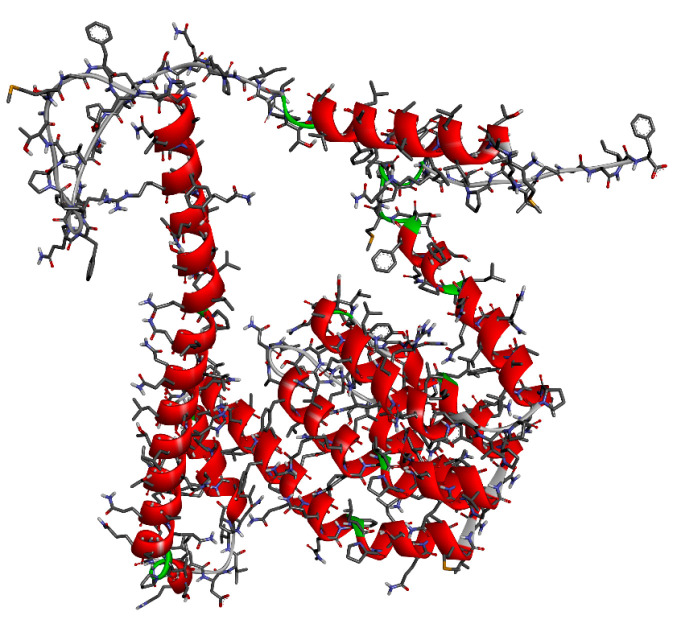
The 3D structure of zein protein downloaded from the European molecular biology laboratory (EMBL-InterPro) with the code Q9SYT3_MAIZE.

In order to elucidate the binding modes of the surfactants within zein protein, a molecular docking simulation was carried out using AutodockVina (La Jolla, CA, USA) [39]. The structure of zein protein (Figure 19) was downloaded from the European molecular biology laboratory (EMBL-InterPro) with the code Q9SYT3_MAIZE [40]. Polar hydrogen atoms have been added to the structure of the built protein for correcting ionization and tautomeric states of amino acid residues [41]. The putative binding sites on zein protein structure were identified using the Discovery Studio Client (version 17.2.0) (Vélizy-Villacoublay, France). All the surfactant ligands were drawn using Chemdraw20.1.1 software [42]. To select the most stable conformation, the geometry of these ligands was subsequently optimized using Molecular Force Field (MMFF94). The ligand and target protein files were converted to the PDBQT format to make it suitable for docking in AutoDock Vina. The interactions of complex protein–ligand conformations were analyzed by Discovery Studio Client software.

### 3.6. Surface Pressure-Area Isotherms

The surface pressure (π) of monolayer isotherms were measured using a Langmuir film balance (KSV Mini Minitrough, Biolin scientific Oy, Espoo, Finland), provided with a Wilhelmy paper plate (KSV) an electrobalance and a through and a barrier system of Teflon. Through and barriers were cleaned following the protocol described in Lozano et al. (2008) [43] Before adding samples, the interface air/aqueous sub-phase was firstly compressed and cleaned by aspiration until the surface pressure was lower than 0.5 mN/m for ensuring the absence of contamination. The concentration, solvents and the proportion between compounds was the following: Surfactant samples were prepared in hexane/ethanol (9:1) at 0.8 mg/mL and Zein samples were prepared at concentration of 0.1905 mg/mL in chloroform/methanol (9:1). The mixtures under study were Zein/surfactant (*v*/*v*, 1/5). Isotherms were recorded at 25 °C and the volume of samples deposited at the interface was 12.5 µL for individual samples and 25 µL for binary samples, using a Hamilton syringe. After 15 min of solvent evaporation, the surface pressure (π) as a function of area (A) was recorded. The rate of barrier symmetric compression was 20 mm/min. The subphase was ultrapure water.

### 3.7. Antimicrobial Activity

#### 3.7.1. Microorganisms and Culture Conditions

The antimicrobial activity of the arginine surfactants based nanoparticles was checked over selected yeasts and bacteria: *Bacilus subtilis* ATCC 6633, *Staphylococcus aureus* ATCC 29213, *Acinetobacter baumanniii* ATCC 19606, *Pseudomonas aeruginosa* ATCC 27853, *Staphylococcus epidermidis* ATCC 12228, *Escherichia coli* ATCC 25922, *Listeria monocytogenes* ATCC 15313 and *Enterococcus faecalis* ATCC 29212, *Candida albicans* ATCC 90028, *Candida jadinni* ATCC 60459, *Candida rugosa* ATCC 10571, *Candida glabrata* ATCC 66032, *Candida paropsilosis* ATCC 22019, *Candida tropicalis* ATCC 7349, *Candida auris* ATCC 21092 and *Candida albicans* ATCC 10231. Frozen stocks of microorganisms were seeded in culture media plates (Müller Hinton agar, MHA, for bacteria and Sabouraud, SBA, for yeasts) and incubated overnight at 35 °C and 30 °C, respectively. Three to four colonies of each strain were dispersed in the appropriate sterile broth (MHB for bacteria and Roswell Park Memorial Institute medium RPMI 1640 for yeasts) to obtain dispersions equivalent to 0.5 in the McFarland turbidity scale (McFarland DEN-1B Grant-bio model densimeter, Shepreth, Cambs, UK). To obtain the bacteria inoculum, the suspensions were diluted to 10 in MHB to reach 10^7^ colony forming units (CFU)/mL. The yeast inoculums were used without dilution (~1.5 × 10^7^ CFU/mL).

#### 3.7.2. Minimum Inhibitory and Minimum Bactericide/Fungicide Concentrations

The minimal inhibitory concentration (MIC), minimal bactericide concentration (MBC) and minimal fungicide concentration (MFC) were determined using the microdilution method according to the CLSI protocol [44]. Each well of a 96-well plate contained 150 μL of sterile broth (MH for bacteria and RPMI 1640 for yeasts). The nanoparticles were serially diluted resulting from 35.6 to 2.225 µg/mL. Surfactant solutions were also prepared in the same manner and used to compare the response to the nanoparticles. The negative control contained sterile broth. Finally, 10 μL of each inoculum was added to the corresponding wells, resulting in a final concentration of 10^6^ UFC/mL. The plates containing bacteria and yeasts were incubated at 30 °C and 37 °C for 24 h, respectively. After the incubation period, the turbidity of wells was checked. The MIC was determined as the lowest concentration without apparent turbidity. To confirm this observation, 20 μL of resazurin at 0.015% *w*/*v* was added to each well and left to react for approximately 1 h for bacteria and 3 h for yeasts at 35° and 30 °C, respectively. After the incubation period, the indicative of bacterial growth, i.e., changing from blue to pink, confirmed the MIC value observed. To obtain the MBC and the MFC, an aliquot of 10 μL of the MIC well and the 2 concentrations immediately above were seeded over agar MH and SB for bacteria and yeasts and incubated for 24 h at 35 and 30 °C, respectively. The MBC and MFC were determined as the lowest concentration in which no colonies were observed on the agar plates.

#### 3.7.3. Hemolytic Activity

Heparinized fresh blood from a white New Zealand rabbit was used to determine the hemolytic activity [45] of the surfactant and the respective nanoparticles. The blood samples were centrifuged for 10 min at 3000 rpm. The supernatant containing the white cells was discarded, while the erythrocytes were suspended in fresh PBS (phosphate buffer solution, pH 7.4). The suspension was centrifuged three times at 3000 rpm for 10 min, in order to eliminate the lysed cells and possible residues, assuring that the analyzed solutions would contain only hemoglobin from the erythrocytes’ residues. After the last washing, a volume of PBS 7.4 equivalent to twice the initial volume was added to dilute the erythrocytes to 8 × 10^9^ cells/mL. To check the quality of the blood 975 μL of ultrapure water and 25 μL of the erythrocytes´ suspension were mixed and gently homogenized for 10 min and centrifuged at 10,000 rpm for 5 min. Finally, the absorbance of the supernatant was measured at λ = 575 nm (Spectrophotometer Shimatzu UV240, Shimatzu^®^, Kyoto, Japan), using PBS 7.4 as the blank.

Before the test, the basal hemolytic activity (0%) was checked using PBS 7.4 as control, while the 100% hemolysis was determined using ultrapure water. The last was used to determine comparatively the individual hemolytic activity (in %). After that, blank and surfactant-loaded zein nanoparticles and their solutions were assayed using the adjusted erythrocytes suspension.

To quantify the leached hemoglobin, the absorbance of the supernatant was measured at λ = 540 nm using PBS 7.4 as blank and the % of hemolysis was calculated according to the following equation:

$$\%\;haemolysis=\frac{Abs_{test\;compound}}{Abs_{Basal\;haemolytic\;activity}\;}\;\times \;100$$

## 4. Conclusions

Zein nanoparticles containing arginine-based surfactants have been prepared using two different methods. Both preparation methods are viable for loading the surfactants into zein nanoparticles The antimicrobial properties and the hemolytic activity of these cationic nanoparticles have been studied. Zein is a protein which offers a great versatility for nanoparticle formation, which allows the obtaining of nanoparticles with different characteristics. The NMR results indicate that when using H_2_O as a co-solvent, there is a binding between the surfactants and the zein mediated by a layer of water molecules that have relative long residence times attached to the hydrophilic surface of the protein (bound water fraction). Qualitatively, the poor transference of saturation from the protein to the surfactants in the STD^off–on^ spectra could reflect either a relative strong binding or a relevant fraction of bound water molecules in the zein binding site. The stability is affected by the type of surfactant loaded, being more notable in Gemini surfactants with longer spacer chains such as C_6_(LA)_2_ and C_9_(LA)_2_. LAM enhanced its antimicrobial activity when nanoencapsulated, while the Gemini surfactants experienced a slightly reduced activity against the bacteria and fungi when loaded in the zein nanoparticles. The nanoencapsulation reduced extensively the hemolytic activity of all surfactants, while the highest reduction was observed in the Gemini surfactants, specially C_6_(LA)_2_. This improves their selectivity when considering the differentiation between microbial and human cell membranes. Therefore, these nanoparticles can become candidates for antimicrobial therapies as a potent activity together with a reduced toxicity which could be achieved after the nanoencapsulation in zein nanoparticles. Those results are in agreement with the NMR, Langmuir monolayers and docking findings, indicating that zein interacts with the surfactants by the aliphatic chain and as a result interferes in the surfactant–lipid interaction necessary for the microbial and cellular interactions. As a result, the cationic charges are freely available to attack and destroy the bacteria and fungi, while the aliphatic chain are not free to disrupt the cellular membranes.

## Data Availability

Not applicable.

## Supplementary Materials

The following supporting information can be downloaded at: https://www.mdpi.com/article/10.3390/ijms24032568/s1.
