# Supplementary material for: Zein Nanoparticles Containing Arginine-Based Surfactants: Physicochemical Characterization and Effect on the Biological Properties
Source: Int J Mol Sci. 2023 Jan 29;24(3):2568. doi: 10.3390/ijms24032568 (PMC9917094; doi:10.3390/ijms24032568)
Supplement: Supplementary file 1 [file ijms-24-02568-s001.zip › ijms-2123865-supplementary.pdf]

Table S1. Composition, size and charge of Blank nanoparticles, LAM, $C_3(LA)_2$, $C_6(LA)_2$ and $C_9(LA)_2$ loaded-zein nanoparticles.

Formulation	Zein (% w/v)	Surfactant (% w/v)	Size DLS (nm)	pdI	Size TEM (nm)	Zeta potential (mV)
**BNp-A**	0.0712	-	294.9	0.066	228	+18.4
**BNp-B**	0.0712	-	267.6	0.05	179	+13.0
**NpLAM-A**	0.0712	0.00712	287	0.048	141	+29.7
**NpLAM-B**	0.0712	0.00712	208.8	0.057	129	+36.8
**Np$C_3(LA)_2$-A**	0.0712	0.00712	341.1	0.204	194	+53.5
**Np$C_3(LA)_2$-B**	0.0712	0.00712	308	0.12	136	+42.3
**Np$C_6(LA)_2$-A**	0.0712	0.00712	200.5	0.068	214	+38.3
**Np$C_6(LA)_2$-B**	0.0712	0.00712	238	0.005	164	+49.9
**Np$C_9(LA)_2$-A**	0.0712	0.00712	180.9	0.029	132	+50.5
**Np$C_9(LA)_2$-B**	0.0712	0.00712	229.1	0.091	121	+49.7

Unloaded nanoparticles (Blank nanoparticles, BNp), method A (A), method B (B). Dynamic light scattering (DLS), Transmission Electron Microscopy (TEM), Polydispersity Index (pdI)

Table S2. ^{1}H Chemical shift (ppm) assignment of samples of the pure molecules LAM, $C_3(LA)_2$, $C_6(LA)_2$ and $C_9(LA)_2$ dissolved in CD_3OD: D_2O 9:1 (v/v) at 25 °C. The numbering scheme of the atoms is given in the structures of Figure 5. The chemical shifts are referenced respect to the TMS signal at 0 ppm.

**Signal LAM**	**^{1}H (ppm)**	**Signal $C_3(LA)_2$**	**^{1}H (ppm)**	**Signal $C_6(LA)_2$**	**^{1}H (ppm)**	**Signal $C_9(LA)_2$**	**^{1}H (ppm)**
**1**	2.161	**1**	1.524	**1**	1.417	**1**	1.399
**3**	4.338	**2**	3.139	**2**	3.093	**2**	3.080
**4**	1.813	**3**	4.203	**3**	4.217	**3**	4.213
**5**	1.638	**4**	1.760	**4**	1.722	**4**	1.724
**6**	3.118	**5**	1.604	**5**	1.586, 1.510	**5**	1.576, 1.504
**8**	1.519	**6**	3.139	**6**	3.122	**6**	3.112
**9**	1.219, 1.188	**7**	2.200	**7**	2.175	**7**	2.168
**10**	3.637	**8**	1.524	**8**	1.510	**8**	1.504
**11**	0.795	**9**	1.230	**9**	1.221	**9**	1.211
**12**	8.231	**10**	1.200	**10**	1.190	**10**	1.185
**13**	Not observed	**11**	1.200	**11**	1.190	**11**	1.185
**14**	Not observed	**12**	0.810	**12**	0.805	**12**	0.795
**15**	7.363	**13**	8.040	**13**	7.985	**13**	7.976
		**14**	8.019	**14**	7.929	**14**	7.884
		**15**	Not observed	**15**	7.180, 7.271	**15**	Not observed
		**16**	7.396	**16**	7.384	**16**	7.374

Table S3. Results of interaction details and docking score in (kJ/mol) of $C_3(LA)_2$ ligand complexed in different binding sites on the surfaces on the zein structures (Q9SYT3_MAIZE).

Docking Score (kJ/mol)	Pocket	Ligand	Receptor Pocket	Interactions type	Interactions category	Distance (Å)
**-6.2**	1	H(NH Amide)	ASN168	Hydrogen Bond	Conventional Hydrogen Bond	2.39496
		H(N^+H Guanidine)	GLN72	Hydrogen Bond	Conventional Hydrogen Bond	2.48701
		C(CH3)	LEU115	Hydrophobic	Alkyl	4.02616
**-6.6**	3	H(N^+H)	VAL166	Hydrogen Bond	Conventional Hydrogen Bond	2.18458
		H(N^+H)	GLN246	Hydrogen Bond	Conventional Hydrogen Bond	2.70272
		C(CH3)	PRO202	Hydrophobic	Alkyl	3.87114
**-6.8**	4	O(C=O)	ASN124	Hydrogen Bond	Conventional Hydrogen Bond	2.41449
		O(C=O)	ALA121	Hydrogen Bond	Carbon Hydrogen Bond	2.9377
**-6**	5	C(CH3)	LEU222	Hydrophobic	Alkyl	4.73127
**-9.2**	6	O(C=O)	GLN131	Conventional Hydrogen Bond	Hydrogen Bond	3.03163
		O(C=O)	GLN176	Conventional Hydrogen Bond	Hydrogen Bond	2.43598
**-8.1**	7	O(C=O)	THR19	Conventional Hydrogen Bond	Hydrogen Bond	2.52101
		H(NH Amide)	THR19	Conventional Hydrogen Bond	Hydrogen Bond	2.21853
		H(NH Amide)	THR19	Conventional Hydrogen Bond	Hydrogen Bond	2.31589

Table S4. Results of interaction details and docking score in (kJ/mol) of $C_6(LA)_2$ ligand complexed in different binding sites on the surfaces on the zein structures (Q9SYT3_MAIZE).

Docking Score (kJ/mol)	Pocket	Ligand	Receptor Pocket	Interactions type	Interactions category	Distance (Å)
**-5.8**	1	H(NH_2 Amide)	GLN76	Conventional Hydrogen Bond	Hydrogen Bond	2.23344
		H(NH_2 Guanidine)	GLN175	Conventional Hydrogen Bond	Hydrogen Bond	2.94159
**-4.4**	2	O(C=O)	SER162	Hydrogen Bond	Carbon Hydrogen Bond	3.62409
		C(CH_3)	PRO119	Hydrophobic	Alkyl	4.98643
**-6.8**	3	H(N+H_2 Guanidine)	GLN246	Hydrogen Bond	Conventional Hydrogen Bond	2.55795
		H(NH_2 Guanidine)	GLN246	Hydrogen Bond	Conventional Hydrogen Bond	2.65436
**-8.5**	4	C(CH_3)	LEU173	Hydrophobic	Alkyl	5.24164
		C(CH_3)	TYR128	Hydrophobic	Pi-Orbitals	5.34872
**-6.1**	5	C(CH_3)	LEU222	Hydrophobic	Alkyl	4.77224
**-8.4**	7	H(NH_2 Amide)	ALA30	Hydrogen Bond	Conventional Hydrogen Bond	2.59322
		H(NH_2 Guanidine)	THR19	Hydrogen Bond	Conventional Hydrogen Bond	2.36816
		C(CH_3)	PHE15	Hydrophobic	Pi-Alkyl	5.15341

Table S5. Results of interaction details and docking score in (kJ/mol) of $C_9(LA)_2$ ligand complexed in different binding sites on the surfaces on the zein structures (Q9SYT3_MAIZE)

Docking Score	Pocket	Ligand	Receptor Pocket	Interactions type	Interactions category	Distance (Å)
**-6.3**	**1**	O(C=O)	ARG218	Hydrogen Bond	Conventional Hydrogen Bond	2.45363
		O(C=O)	ARG218	Hydrogen Bond	Conventional Hydrogen Bond	2.6101
		H(N^+H Guanidine)	GLU226	Hydrogen Bond	Conventional Hydrogen Bond	1.9814
**-3.5**	**2**	C(CH_2 Spacer)	ASN192	Carbon Hydrogen Bond	Hydrogen Bond	3.38474
**-6.4**	**3**	H(N^+H Guanidine)	GLN205	Hydrogen Bond	Conventional Hydrogen Bond	2.72788
		H(N^+H Guanidine)	GLN205	Hydrogen Bond	Conventional Hydrogen Bond	2.831
		C(CH_2 Spacer)	ASN168	Hydrogen Bond	Carbon Hydrogen Bond	3.06885
**-7.1**	**4**	H(N^+H Guanidine)	GLN133	Hydrogen Bond	Conventional Hydrogen Bond	2.21917
		C(CH_3)	LEU134	Hydrophobic	Alkyl	5.10831
		C(CH_3)	LEU159	Hydrophobic	Alkyl	5.41502
**-7.2**	**6**	O(C=O)	GLN180	Hydrogen Bond	Conventional Hydrogen Bond	2.93727
		H(NH Amide)	GLN135	Hydrogen Bond	Conventional Hydrogen Bond	2.39441
**-8**	**7**	H(NH Amide)	PHE22	Hydrogen Bond	Pi-Donor Hydrogen Bond	3.01253

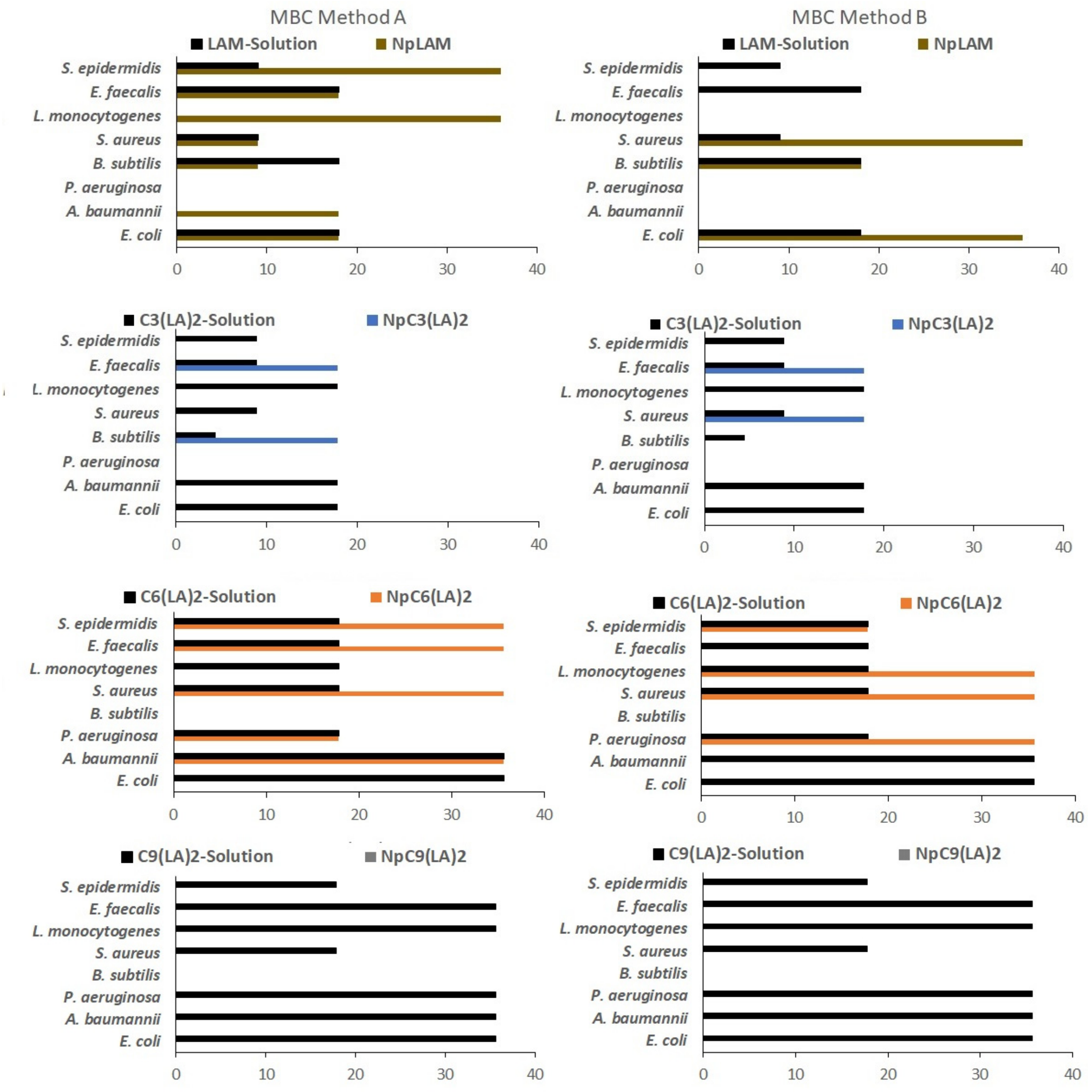

Figure S1. Determination of the MBC (µg/ml) for LAM, $C_3(LA)_2$, $C_6(LA)_2$, $C_9(LA)_2$ solutions and loaded-zein nanoparticles prepared by methods A and B against bacteria.

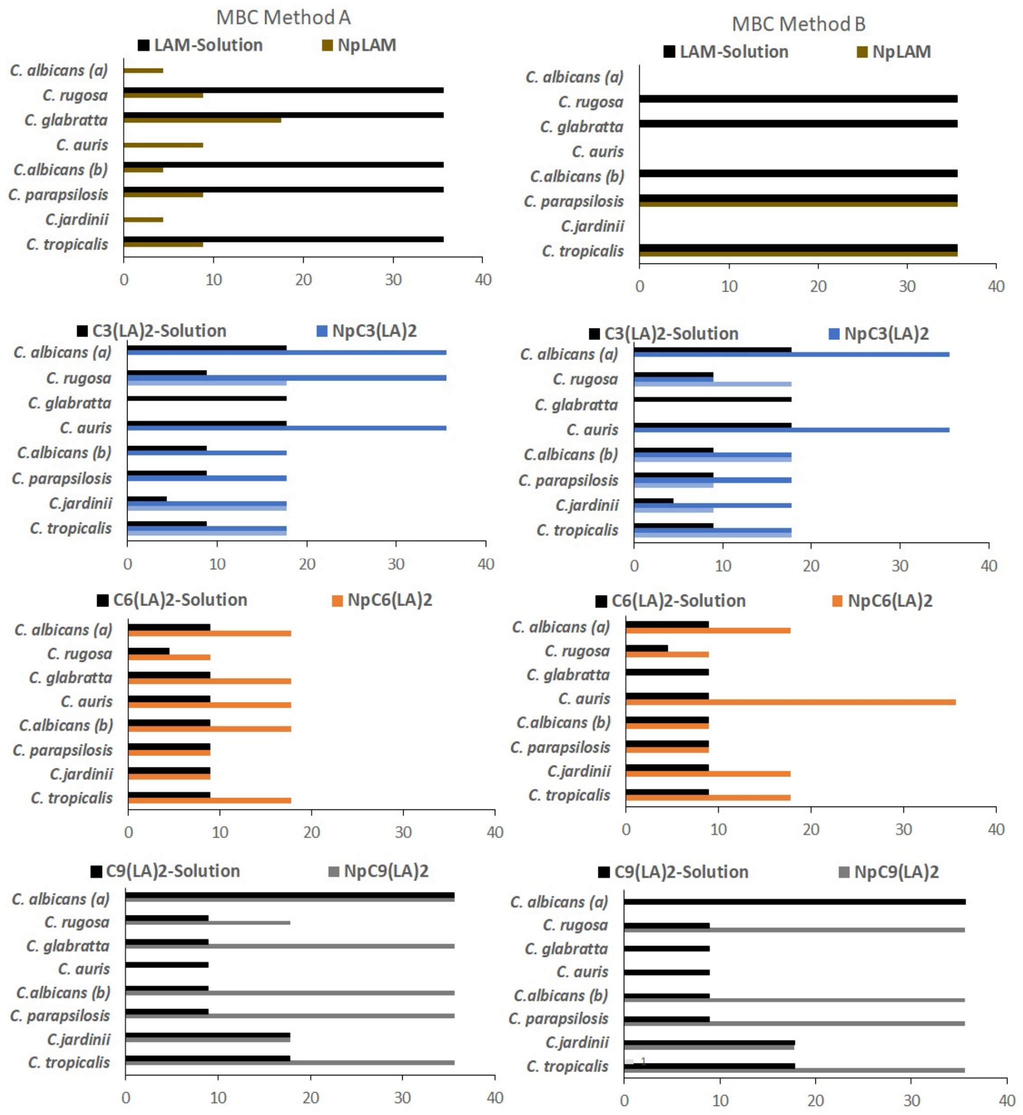

Figure S2. Determination of the MFC (μg/ml) for LAM, $C_3(LA)_2$, $C_6(LA)_2$, $C_9(LA)_2$ solutions and loaded-zein nanoparticles prepared by methods A and B against yeasts.
